# Human uridine 5′-monophosphate synthase stores metabolic potential in inactive biomolecular condensates

**DOI:** 10.1016/j.jbc.2023.102949

**Published:** 2023-01-25

**Authors:** Deborah M. Kim-Holzapfel, Raja Dey, Brian C. Richardson, Danushka Arachchige, Kanamata Reddy, Humberto De Vitto, Janarjan Bhandari, Jarrod B. French

**Affiliations:** 1 Department of Biochemistry and Cell Biology, Stony Brook University, Stony Brook, New York, USA; 2 Molecular and Cellular Biology PhD Program, Stony Brook University, Stony Brook, New York, USA; 3 The Hormel Institute, University of Minnesota, Austin, Minnesota, USA

**Keywords:** Nucleotide metabolism, mesoscale assembly, membraneless organelles, metabolic potential, pyrimidines, purinosome, AEX, anion exchange chromatography, AUC, analytical ultracentrifugation, EM, electron microscopy, HsUMPS, Human UMP synthase, LC/MS, liquid chromatography, mass spectrometry, MBP, maltose binding protein, OMP, orotidine 5′-monophosphate, OMPDC, orotidine 5′-monophosphate decarboxylase, OPRT, orotate phosphoribosyltransferase, SEC, size-exclusion chromatography, TEV, tobacco etch virus, UMP, uridine 5′-monophosphate

## Abstract

Human uridine 5′-monophosphate synthase (HsUMPS) is a bifunctional enzyme that catalyzes the final two steps in *de novo* pyrimidine biosynthesis. The individual orotate phosphoribosyl transferase and orotidine monophosphate domains have been well characterized, but little is known about the overall structure of the protein and how the organization of domains impacts function. Using a combination of chromatography, electron microscopy, and complementary biophysical methods, we report herein that HsUMPS can be observed in two structurally distinct states, an enzymatically active dimeric form and a nonactive multimeric form. These two states readily interconvert to reach an equilibrium that is sensitive to perturbations of the active site and the presence of substrate. We determined that the smaller molecular weight form of HsUMPS is an S-shaped dimer that can self-assemble into relatively well-ordered globular condensates. Our analysis suggests that the transition between dimer and multimer is driven primarily by oligomerization of the orotate phosphoribosyl transferase domain. While the cellular distribution of HsUMPS is unaffected, quantification by mass spectrometry revealed that *de novo* pyrimidine biosynthesis is dysregulated when this protein is unable to assemble into inactive condensates. Taken together, our data suggest that HsUMPS self-assembles into biomolecular condensates as a means to store metabolic potential for the regulation of metabolic rates.

Pyrimidine nucleotides are essential for all forms of life. They play critical roles in the transfer of genetic information, cell signaling, cell membrane assembly, and as both substrates and cofactors in numerous biosynthetic pathways (1, 2, 3, 4, 5). The concentration of cellular pyrimidine nucleotide pools is maintained through the operation of two complementary pathways. A single-step salvage, or recycling, pathway generates pyrimidine nucleotides from a ribose sugar and preformed base, while the *de novo* biosynthetic pathway generates pyrimidines over several steps, using amino acids and other metabolites as building blocks. A small number of mostly parasitic species lack the *de novo* biosynthetic pathway and rely exclusively on salvage to meet their pyrimidine needs (6). In most other organisms, the relative contribution of salvage and *de novo* depends upon cell type and stage of development. Biosynthesis of pyrimidines by the *de novo* pathway is more tightly controlled and, like purine metabolism, is known to be upregulated in cancers and other rapidly dividing cells (7, 8, 9, 10, 11). As a result, the enzymes of this pathway are clinically validated targets for antimetabolites that treat a number of human diseases, including several forms of cancer (5, 12).

The six chemical transformations involved in the biosynthesis of uridine 5′-monophosphate (UMP) make use of up to six enzymes (Fig. 1). While bacteria typically employ six separate proteins, in humans, these reactions are catalyzed by only three gene products, a trifunctional protein, carbamoyl-phosphate synthetase 2, aspartate transcarbamoylase, and dihydroorotase, a monofunctional protein, dihydroorotate dehydrogenase (DHODH), and a bifunctional protein, uridine monophosphate synthase (UMPS). The two multifunctional enzymes are found predominantly in the cytoplasm, while DHODH is an inner mitochondrial membrane protein (3, 4, 5). Both carbamoyl-phosphate synthetase 2, aspartate transcarbamoylase, and dihydroorotase and UMPS have also been reported to localize in the nucleus under some conditions (13). The role of these enzymes in the nucleus, however, is still unclear.

**Figure 1:**
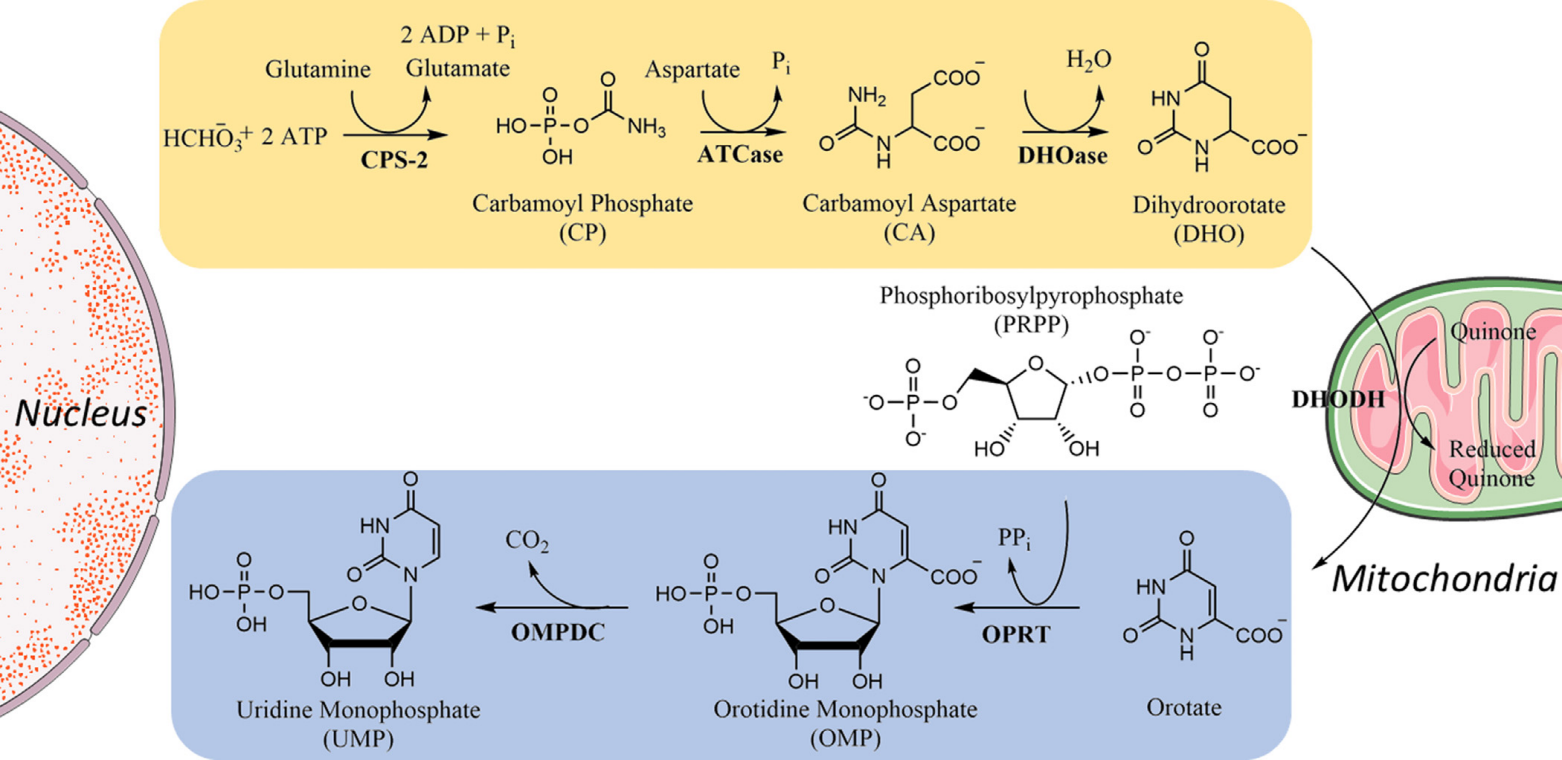
***De novo* biosynthesis of pyrimidines.** Biosynthesis of UMP takes place in six steps and is catalyzed by three enzymes in humans. The first three transformations that put together carbonate, ATP, the nitrogen from glutamine, and aspartate to yield dihydroorotate, are catalyzed by the enzyme CAD. CAD is composed of three enzymatic domains including carbamoyl phosphate synthase II (CPS-II), aspartic transcarbamoylase (ATCase, aspartate carbamoyl transferase), and dihydroorotase (DHOase). The next step in the pathway is catalyzed by dihydroorotate dehydrogenase (DHOD), which is the only mitochondrial protein in the pathway. The final two chemical transformations are catalyzed by UMP synthase (UMPS), which is composed of an orotate phosphoribosyltransferase (OPRT) domain and an orotidine monophosphate decarboxylase (OMPDC) domain. CAD, carbamoyl-phosphate synthetase 2, aspartate transcarbamoylase, and dihydroorotase; UMP, uridine 5′-monophosphate.

UMPS is a two-domain enzyme that catalyzes the final two steps in the *de novo* biosynthesis of UMP. It is composed of the transferase, orotate phosphoribosyl transferase (OPRT), that adds the orotate base to the ribose ring of phosphoribosyl pyrophosphate (PrPP) to produce orotidine 5′-monophosphate (OMP), and a decarboxylase, orotidine monophosphate decarboxylase (OMPDC), that decarboxylates the product of the OPRT-catalyzed reaction to yield the final product, UMP (Fig. 1). The OMPDC enzyme has been broadly studied because of the unusually high catalytic rate enhancement that it provides (14, 15). OMPDC is an obligate dimer, with residues from both protomers shaping the active site (14, 15, 16). The OPRT domain is also found predominantly as a dimer but has been shown to undergo substrate-dependent conformational changes that drive a functionally important monomer to dimer transition (17, 18, 19). The human full-length UMPS enzyme has been reported to have multiple oligomeric states, including a dimeric, tetrameric, and other multimeric species (20, 21, 22). The functional significance of the different oligomeric states is, as yet, unknown. Crystal structures are available for both the OMPDC and OPRT domains of the human enzyme; however, no structural information is available for the full-length UMPS protein.

Recent findings in a wide variety of different organisms have demonstrated the functional relevance of biomolecular condensates or mesoscale assemblies of proteins implicated in a wide variety of cellular processes from cell wall synthesis, controlling cell shape, response to cell stress, regulation of gene expression, and many others (23, 24, 25, 26, 27). There are an increasing number of examples of this phenomenon, including several that play specific roles in regulating metabolic pathways (28, 29, 30, 31). In nucleotide metabolism, specifically, higher order structures of proteins have been observed for both purine and pyrimidine biosynthetic proteins (28, 29, 32, 33, 34, 35). The proteins of the purine biosynthetic pathway come together to form the purinosome, an assembly that can promote channeling of intermediates, improve flux, and selectively localize within cells (36, 37, 38, 39, 40, 41). Much less is known about multiprotein structures of the proteins of pyrimidine biosynthesis, and there is, as yet, no evidence of a purinosome-like assembly of all of the pyrimidine biosynthetic proteins. The physical separation of the enzymes, with DHODH located in mitochondrial membranes, suggests that this type of structure is unlikely to be formed. However, individual proteins involved in downstream pyrimidine metabolism have been seen to assemble into higher order structures, including cytidine triphosphate synthase filaments as well as both rod and ring-shaped structures of inosine 5′-monophosphate dehydrogenase (32, 34, 42, 43).

Here, we report that human UMPS (HsUMPS) is found in a dynamic equilibrium between at least two structurally and functionally distinct states. The distribution between these states is sensitive to perturbations of the active site and to the presence of substrates. The smaller species is a dimer with an S-shaped structure, while the larger species is a polymeric, globular condensate that is enzymatically inactive. While the oligomerization of HsUMPS does not appear to alter overall cellular distribution, analysis of UMP biosynthetic rate by mass spectrometry suggests that a mutant HsUMPS that is unable to polymerize shows unregulated high activity. From our analysis of structure, activity, spatial localization, and metabolic flux, we propose that the larger, aggregated form of UMPS is likely a means to store metabolic potential in order to quickly respond to fluctuations in pyrimidine nucleotide concentrations.

## Results and discussion

### HsUMPS equilibrates between two structurally different states

HsUMPS protein was expressed and purified as a fusion to maltose binding protein (MBP) to improve expression yields and to assist purification. The protein expressed well as an MBP fusion, yielding 2 to 10 mg/l of terrific broth. It was purified using a four step, three column process involving affinity chromatography using amylose resin, on-column tobacco etch virus (TEV) cleavage of the 6His-MBP-UMPS protein, removal of uncleaved protein, and TEV protease by Ni-NTA IMAC, followed by a polishing step using size-exclusion chromatography (SEC) or anion exchange chromatography (AEX). The resulting protein ran as a single band on SDS-PAGE and was estimated to be >95% pure.

**Figure 2:**
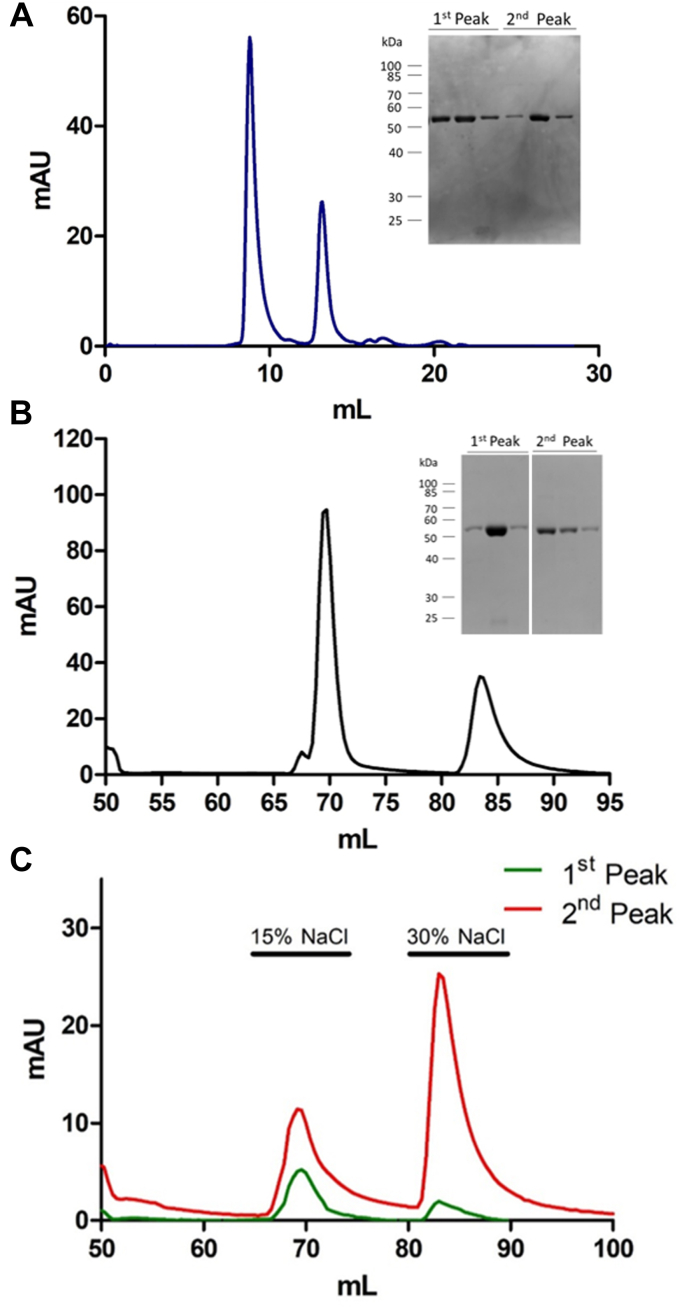
**HsUMPS has two structural states that slowly equilibrate.***A*, size-exclusion chromatography of HsUMPS yields a chromatogram with two distinct peaks (*A*), both of which are the HsUMPS protein (*A*, inset). *B*, similarly, two peaks are also observed when the protein is subjected to anion exchange chromatography. *C*, when a sample taken from either of the isolated peaks is re-analyzed chromatographically, the same two peaks are observed. In the figure, % NaCl refers to percentage of a 1 M solution of NaCl (15% = 150 mM). Note that in the gel figure in (*B*, inset) the two sections are from the same gel—two intervening lanes were removed for clarity. HsUMPS, human UMP synthase; UMP, uridine 5′-monophosphate.

During the purification process, we consistently observed two peaks from SEC of HsUMPS (Fig. 2*A*). One of these peaks corresponded to a larger molecular weight (MW) species with a retention time well after the established void volume of our column, while the second, smaller MW peak, is consistent with a dimer of HsUMPS. Despite the observation of a bimodal distribution in the SEC, the protein contained in both peaks was clearly HsUMPS (Fig. 2*A*, inset). We observed a similar distribution of the protein when we repeated this experiment using AEX (Fig. 2*B*). In this case, we presume that the order of elution is reversed, with the larger MW form likely to be more highly charged and thus elute later than the lower MW form in AEX. This was later confirmed by activity assays (see below). To determine if these distinct species were stable and separable, we collected the protein eluting at each peak and reran the chromatographic separation. In both cases, we observed the same bimodal distribution (Fig. 2*C*). These results suggest that HsUMPS exists in at least two distinct, structurally well-defined states that exist together in equilibrium.

### The two structural states of HsUMPS have different levels of activity

**Figure 3:**
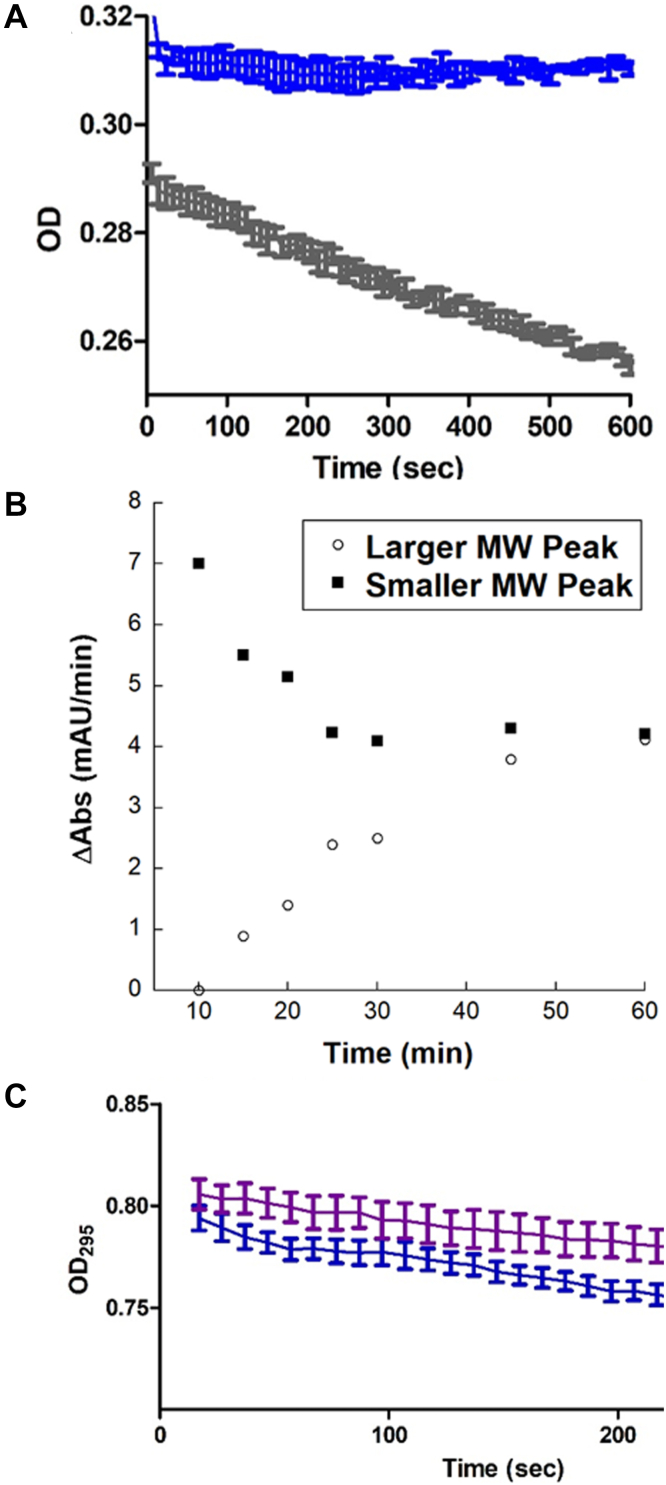
**The two distinct structural states of HsUMPS had different levels of enzyme activity.***A*, the initial rates of reaction for the two different MW species HsUMPS, immediately after SEC, were determined spectrophotometrically. The larger molecular weight species (*blue*) appeared to be initially inactive, while the smaller molecular weight species (*gray*) showed robust activity. *B*, initial rates for both samples were measured at intervals over 60 min. Both isolated species appear to completely re-equilibrate after approximately 60 min. The smaller, more active, species appears to return to equilibrium somewhat more quickly than the larger molecular weight form of HsUMPS. *C*, when incubated with the substrate, orotate, for 10 min prior to the first initial rate measurement, both the smaller species (*blue*) and the larger species (*purple*) appear to be active with similar rates of turnover. For *A* and *C*, an average of triplicate measurements is plotted, and the error bars show the standard error for each measurement. HsUMPS, human UMP synthase; MW, molecular weight; UMP, uridine 5′-monophosphate.

UMPS converts the substrates orotate and PrPP into UMP using two reactions sequentially catalyzed by the OPRT and OMPDC domains (Fig. 1). OPRT is a transferase that adds the orotate base to the ribose sugar, generating the intermediate OMP. OMP is then decarboxylated by OMPDC, yielding the final product, UMP. Of the two enzyme activities, the OPRT-catalyzed reaction is rate limiting (4, 44, 45, 46). Using established spectrophotometric assays, we determined the steady-state kinetic parameters of both the OPRT (*K*_*m*_ = 2.0 ± 0.2 μM, and *k*_*cat*_ = 0.74 s^−1^) and OMPDC (*K*_*m*_ = 8.6 ± 1 μM, and *k*_*cat*_ = 1.9 s^−1^) domains of the wildtype HsUMPS that we expressed (Fig. S1). These values are consistent with the previously measured kinetics of HsUMPS (15, 47, 48, 49). Note that these experiments were conducted with purified enzyme that presumably contained an equilibrium mixture of the two different structural states. To determine if the different HsUMPS species had different activities, we first separated the two populations of the enzyme by SEC and immediately measured initial rates. Surprisingly, while the smaller MW species exhibited an initial rate of reaction similar to what we had observed for the mixture, the larger species was effectively inactive (Fig. 3*A*). By measuring the initial reaction rate of the separated species over several time points, we determined that larger MW equilibrates after approximately 60 min, while the more active, smaller MW species returns to equilibrium somewhat faster (Fig. 3*B*). Another important observation from these data is that the OPRT domain of HsUMPS appears to be fully active as a monomer and does not appear to be an obligate dimer like OMPDC or OPRT from some other species (17, 18). This assumes that the HsUMPS dimer is formed *via* dimerization at the OMPDC domain and does not involve interactions between OPRT domains, an observation later confirmed by our structural analysis described further below.

### Distribution of species depends upon activity and presence of substrate

**Figure 4:**
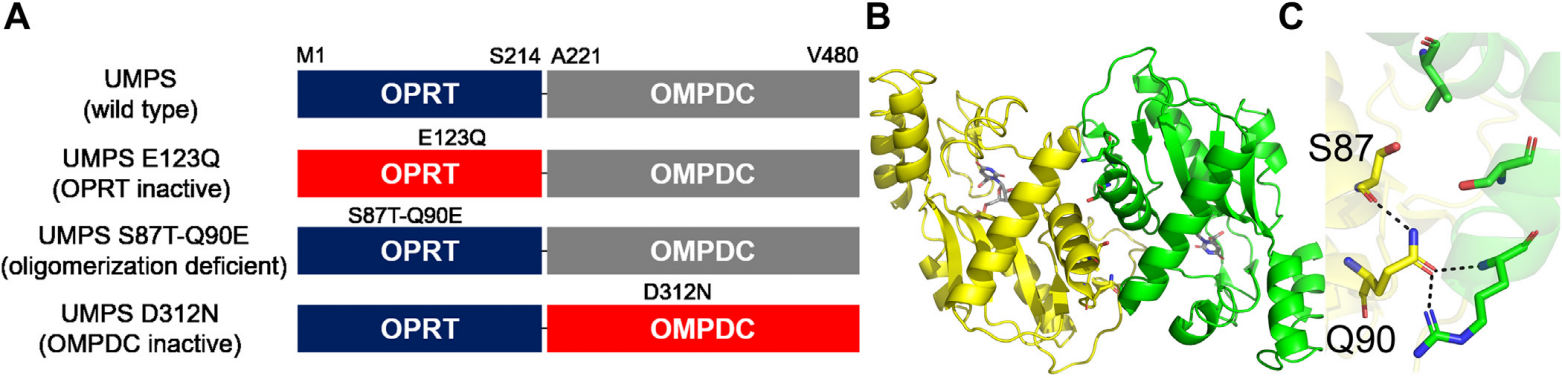
**UMPS domains and mutants.***A*, the two domains of UMPS are shown above with sequence numbers given to delineate the start and end points of the domains. The two native domains are shown in *blue* (OPRT) and *gray* (OMPDC), while domains with mutants are shown colored *red*. The specific mutations are shown above each domain at the approximate position in the sequence. Note that both the E123Q and D312N mutants are active site mutants of the OPRT and OMPDC domains, respectively. *B*, the structure of the OPRT dimer is shown (*B*) with the mutated residues shown as *sticks* (the OMP substrate is also given in grey to show the position of the active site). *C*, a zoomed in view of the location of the site (*C*) shows the mutated residues and the intraprotein and interprotein H-bonding interactions made. OMP, orotidine 5′-monophosphate; OMPDC, orotidine 5′-monophosphate decarboxylase; OPRT, orotate phosphoribosyltransferase; UMP, uridine 5′-monophosphate.

**Figure 5:**
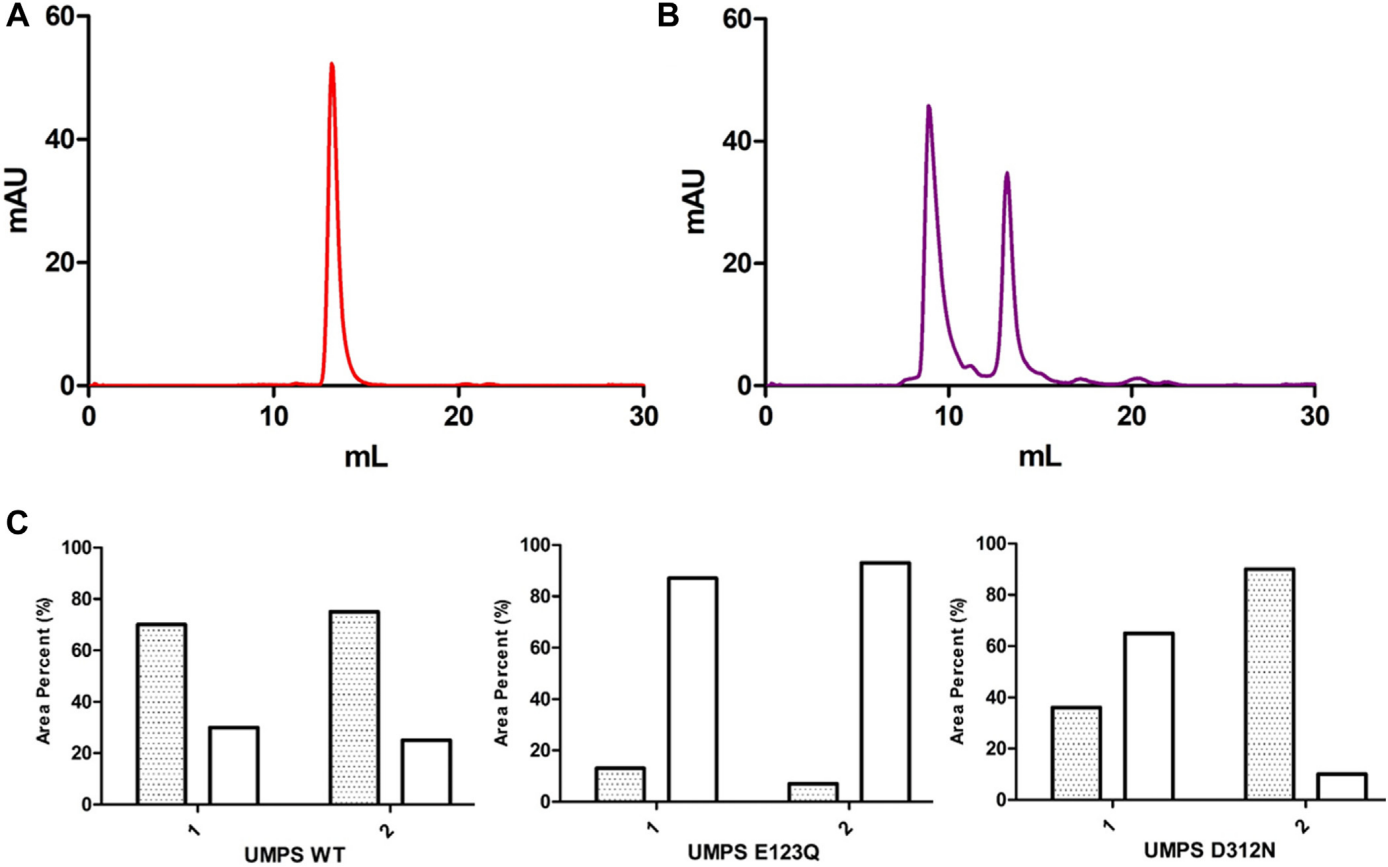
**The distribution between the different forms of HsUMPS is sensitive to the presence of orotate and to changes in the OPRT active site.***A* and *B*, SEC analysis of active site mutants of HsUMPS showed that the OPRT mutant (*A*, E123Q, *red curve*) was present as a single, lower molecular weight, species, while the OMPDC mutant (*B*, D312N, *purple curve*) had a similar distribution as the native protein. *C*, when the results of SEC for the three variants of HsUMPS were compared in the presence and absence of the substrate, orotate (*C*, 1: buffer only, 2: buffer with 500 μM orotate), there was a significant shift in the distribution of species for the OMPDC mutant, while little change was observed for the other two. Note that the bar graphs in *C* show the integrated volume under the curve for each peak under the conditions listed. HsUMPS, human UMP synthase; OMPDC, orotidine 5′-monophosphate decarboxylase; OPRT, orotate phosphoribosyltransferase; SEC, size-exclusion chromatography; UMP, uridine 5′-monophosphate.

To further investigate the distribution of, and transition between, HsUMPS states, we conducted a similar analysis on active site mutants. We expressed and purified an OPRT mutant, E123Q, and an OMPDC mutant, D312N (Fig. 4*A*). These active-site mutants are known to inactivate the enzyme while maintaining the overall structure of the domain (15, 18). Prior to our analysis, we first confirmed that both mutants retained activity in the nonmutated domain and exhibited similar kinetics to the wildtype (Fig. S2). While the SEC analysis indicated that the distribution between the two states of HsUMPS for the OMPDC mutant (UMPS D312N) essentially mirrored that of the wildtype (Fig. 5*A*), the OPRT mutant (UMPS E123Q) showed a single peak corresponding to the lower MW species (Fig. 5*B*). We extended this analysis by looking at the peak distribution in the presence of 500 μM orotate, a substrate of OPRT. The presence of orotate did not alter the distribution for either the native or OPRT mutant (Fig. 5*C*, wildtype [WT] and UMPS E123Q). The OMPDC mutant, however, showed a significant shift toward the larger state in the presence of orotate (Fig. 5*C*; UMPS D312N). Similarly, we examined the effect of orotate on the kinetics of the different states. When we preincubated the two different species with orotate for 10 min prior to measuring the kinetics, the initial rates of both were quite similar (Fig. 3*C*). This indicated that the presence of substrate, or the intermediate OMP, significantly accelerated the transition from the larger, inactive state to the active state. These experiments suggest that the OPRT domain is predominantly responsible for driving the transition between the two different structural states of HsUMPS. The presence of orotate, or the product of the OPRT-catalyzed reaction, OMP can shift the equilibrium toward the smaller, active, species, as evidenced by the kinetics results. Conversely, a build-up of orotate or OMP, as would be the case for the OMPDC mutant, drives the distribution toward the inactive state.

### Size, shape, and oligomerization of HsUMPS

Based on a set of MW standards run on SEC using the same conditions as for HsUMPS (Fig. S3), the smaller MW species of HsUMPS eluted with a predicted MW of approximately 65 kDa, while the larger species eluted at a volume consistent with a MW of approximately 500 kDa. As the HsUMPS protomer has an expected MW of 52 kDa, the SEC results indicate that the lower MW peak observed in SEC represents a monomer or dimer. OMPDC is known to be an obligate dimer, suggesting that UMPS is likely to be observed in this form. To more clearly define the oligomerization and overall structure of HsUMPS, we conducted a series of biophysical analyses on the native and mutant forms of the protein. As most of our data indicated that the OPRT domain was involved in promoting the assembly of the larger HsUMPS structure, we first generated an OPRT mutant that would be unable to dimerize through the OPRT domain. Using the existing crystal structure of the OPRT domain from HsUMPS, we generated a double mutant, S87T/Q90E, that introduced steric and electronic clashes at the native OPRT dimer interface (Fig. 4, *B* and *C)*. The mutant, which we refer to as the OPRT dimerization–deficient mutant, retained the enzyme activity at both OPRT and OMPDC domains (Fig. S4). Analysis by SEC (Fig. S5) indicates that this mutant appears monodisperse and behaves similarly to the OPRT active site mutant.

**Figure 6:**
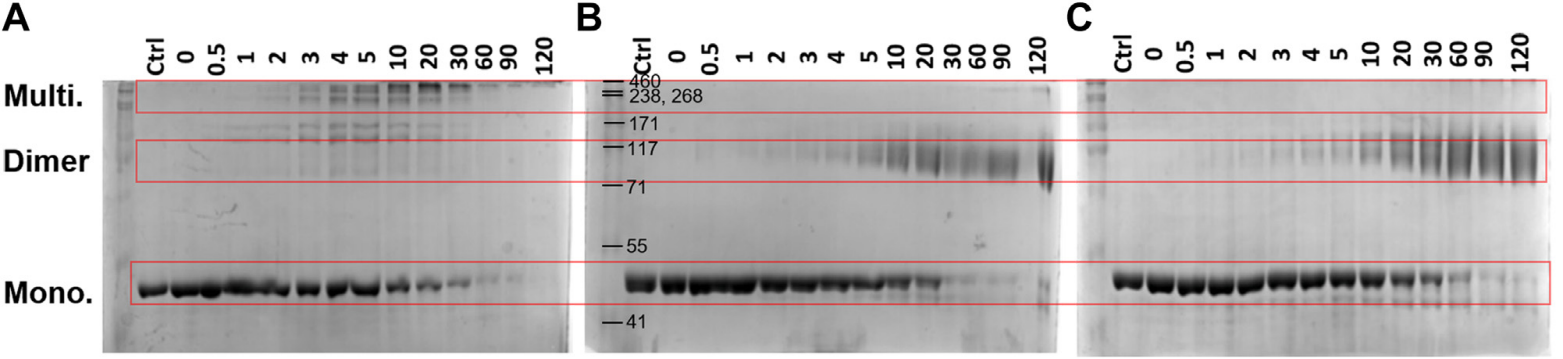
**Crosslinking of HsUMPS.***A*–*C*, native UMPS (*A*), the OPRT active site mutant (*B*), and the OPRT dimerization-deficient mutant (*C*) were treated with glutaraldehyde and analyzed by SDS-PAGE over time. For the native protein, several higher molecular weight multimers were visible within a few minutes after beginning the reaction. After 2 h, the protein was almost completely aggregated into large molecular weight forms. Conversely, for the two OPRT mutants, a species with a molecular weight consistent with a dimer was observed to be the predominant form, even after crosslinking for 2 h. The molecular weight markers are the same on all three gels and the corresponding weights are shown in *B*. The *red boxes* delineate likely monomers (Mono, expected MW 52 kDa), dimers (Dimer, expected MW 104 kDa), and multimers (Multi.). HsUMPS, human UMP synthase; OPRT, orotate phosphoribosyltransferase; UMP, uridine 5′-monophosphate.

Using this dimerization-deficient mutant, we first conducted a series of aldehyde crosslinking experiments. The native, OPRT active site mutant, and OPRT dimerization-deficient mutant were all incubated with glutaraldehyde over a range of time and analyzed by SDS-PAGE to visualize the progression of cross-linking (Fig. 6). For the native enzyme, a small amount of dimer and intermediate sized species were observed in the first 10 min, but the predominant species after 30 min of incubation was a high MW multimer (Fig. 6*A*). Conversely, for both the OPRT mutants, the dimer was the predominant species observed after crosslinking for up to 2 h (Fig. 6, *B* and *C*). These data are consistent with the chromatography results, suggesting that the OPRT mutants exist primarily as a dimer, while the native enzyme is prone to oligomerization. In addition to the crosslinking analysis, we used analytical ultracentrifugation (AUC) to confirm the likely MW of our samples. For the OPRT active-site mutant, which is monodisperse by SEC and is expected to be predominantly the lower MW species, analysis of AUC data by SEDFIT gave a predicted MW of 102 kDa, consistent with a dimer (Fig. S6). Similarly, analysis of the AUC data for the OPRT dimerization-deficient mutant yielded a clear peak consistent with a dimer (104 kDa). For the native HsUMPS enzyme, consistent with our SEC results, the sedimentation analysis suggested that while the dimer is the predominant form, multiple different MW species were present (Fig. S6).

**Figure 7:**
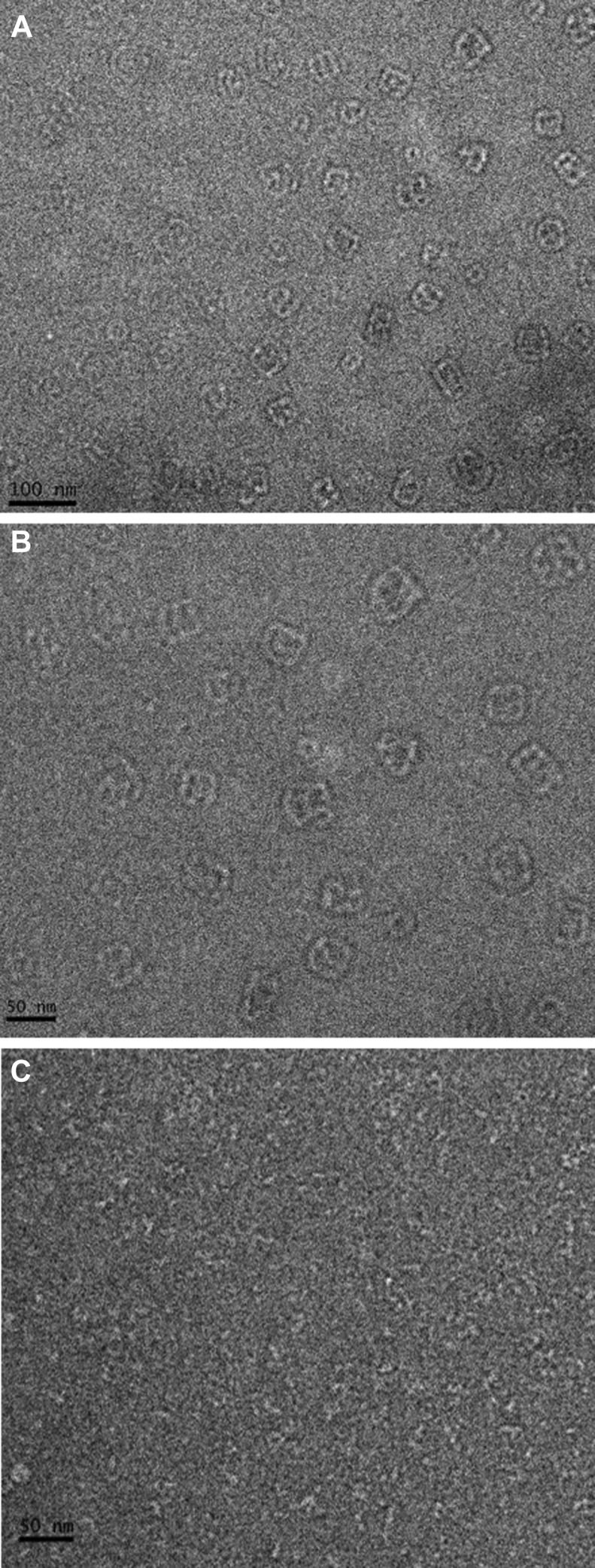
**Structure of higher molecular weight form of HsUMPS.***A* and *B*, negative-stain electron microscopy of native HsUMPS protein at 30,000× magnification (*A*) and at 49,000× magnification (*B*) shows a reasonably well defined structure of HsUMPS. The 30 to 60 nm structures appear to be linear polymers that coalesce into approximately globular structures. Images of the dimerization-deficient mutant, S87T/Q90E (*C*), show no evidence of an HsUMPS polymer. HsUMPS, human UMP synthase; UMP, uridine 5′-monophosphate.

**Figure 8:**
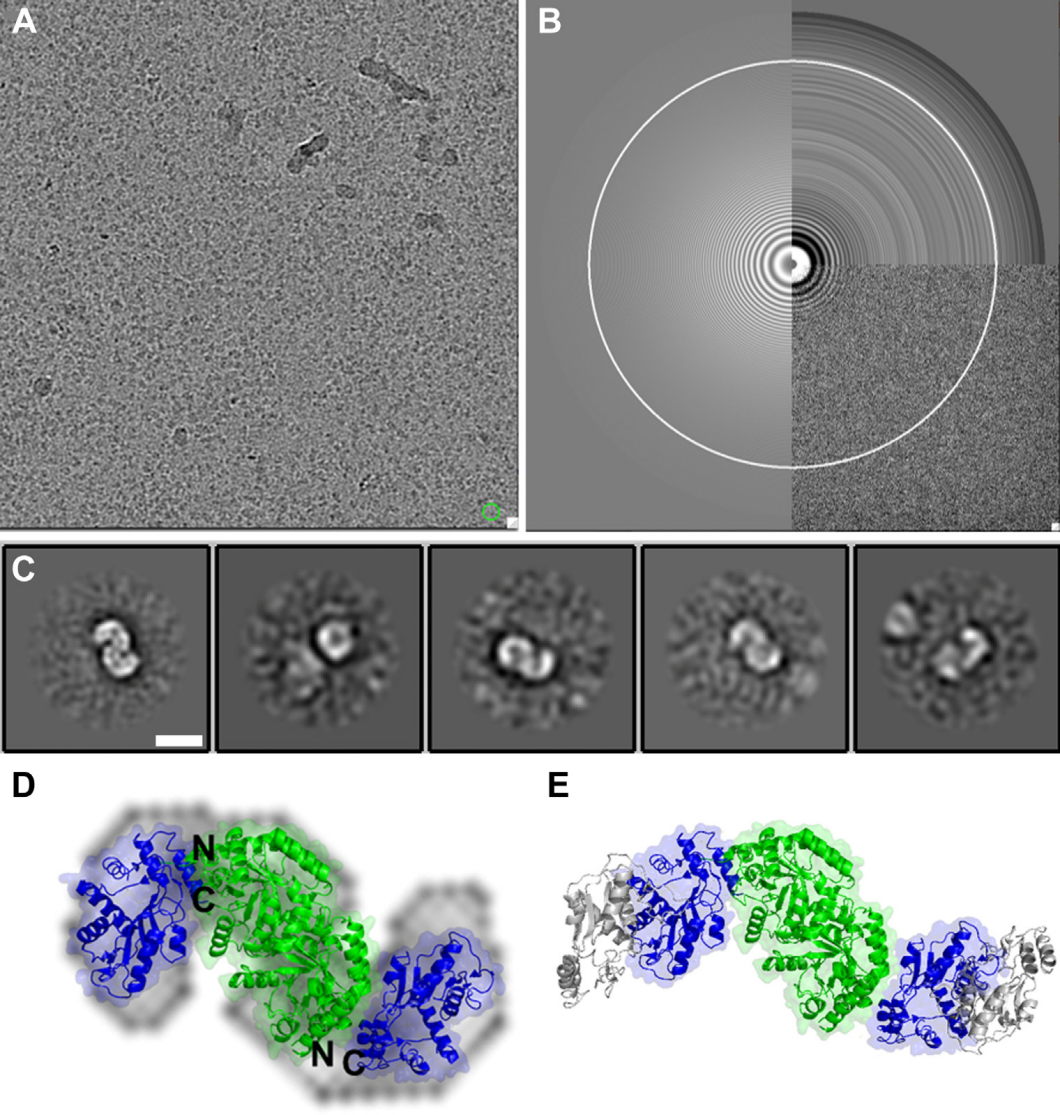
**Single particle cryo-electron microscopy of E123Q UMPS.***A* and *B*, a representative motion corrected micrograph from a sample of the OPRT active site mutant of UMPS (E123Q) shows a good distribution of particles (*A*, defocus −2.4, circle diameter 100 Å). The CTF fit of the micrograph in (*A*) is shown in *B*. *C*, the preliminary 2D classification from ∼20,000 particles shows an obvious S-shaped structure (the five classes shown represent about 3700 particles—additional 2D classes from a larger dataset are shown in Fig. S7). A model of HsUMPS was manually built into the 2D class (*D*) using the known crystal structures of HsOMPDC (*green*, 2JGY) and HsOPRT (*blue*, 2WNS). The position of the N terminus of the OPRT domains and the C terminus of the OMPDC domains are shown on the figure as N and C, respectively. *D* and *E*, the orientation of the domains shown in (*D*) allows for dimerization of OPRT (*E*, additional OPRT protomer given in *gray*). Scale bar in (*C*) is 50 Å, and the box size is 256 pixels at 0.89 Å/pixel. HsUMPS, human UMP synthase; OMPDC, orotidine 5′-monophosphate decarboxylase; OPRT, orotate phosphoribosyltransferase; UMP, uridine 5′-monophosphate.

To further characterize the structure of HsUMPS, we used both negative stain and cryo-electron microscopy (cryo-EM). Negative stain EM of the native protein revealed the presence of large, globular structures with an approximate size of 40 to 60 nm (Fig. 7, *A* and *B*). These structures, which appear to be comprised of strands of linear polymers, show some variation in size and overall shape but appear to be relatively narrowly distributed in this regard. The size and distribution of these particles is consistent with our observation of a large, relatively well structured, UMPS polymer. Some evidence of a smaller species was also visible in the negative stain images; it was difficult, however, to determine the overall structure of the smaller species with these samples. Negative stain EM of the OPRT active site mutant (E123Q) or OPRT dimerization-deficient mutant (S87T/Q90E) showed no evidence of a larger species. Smaller particles of varying size and shape, approximately 5 to 10 nm in size, were observed for these samples (Fig. 7*C*). We then sought to characterize the structure of HsUMPS at higher resolution using single particle cryo-EM. Despite several attempts with the native HsUMPS protein, under varying conditions, we were unable to identify a sufficient quantity of particles to characterize the larger species. We attribute this to its variability in size and inherent structural flexibility. Using the E123Q OPRT mutant, however, we were able to see clear, distinct particles in the micrographs for the smaller MW species (Fig. 8). 2D-classification of these particles yielded preliminary 2D classes that showed an S-shaped structure (Figs. 8*C* and S7). Due to orientation bias of the protein on the grids and the high degree of interdomain flexibility of the OPRT domain relative to the OMPDC domain, we were unable to identify enough particle orientations to generate 3D maps of sufficient quality to build a model. Despite this limitation, we used the existing crystal structures of the separate HsOMPDC and HsOPRT domains, along with the *L. donovannii* UMPS structure to produce an overall model for the HsUMPS structure (Fig. 8, *D* and *E*) (15, 17). In addition, we collected SEC–small angle X-ray scattering data on the small MW peak. The models fit to these data are consistent with an approximately S-shaped dimer that has some degree of interdomain flexibility (Fig. S8).

### Effects of HsUMPS biomolecular condensates on cellular localization and metabolic flux

**Figure 9:**
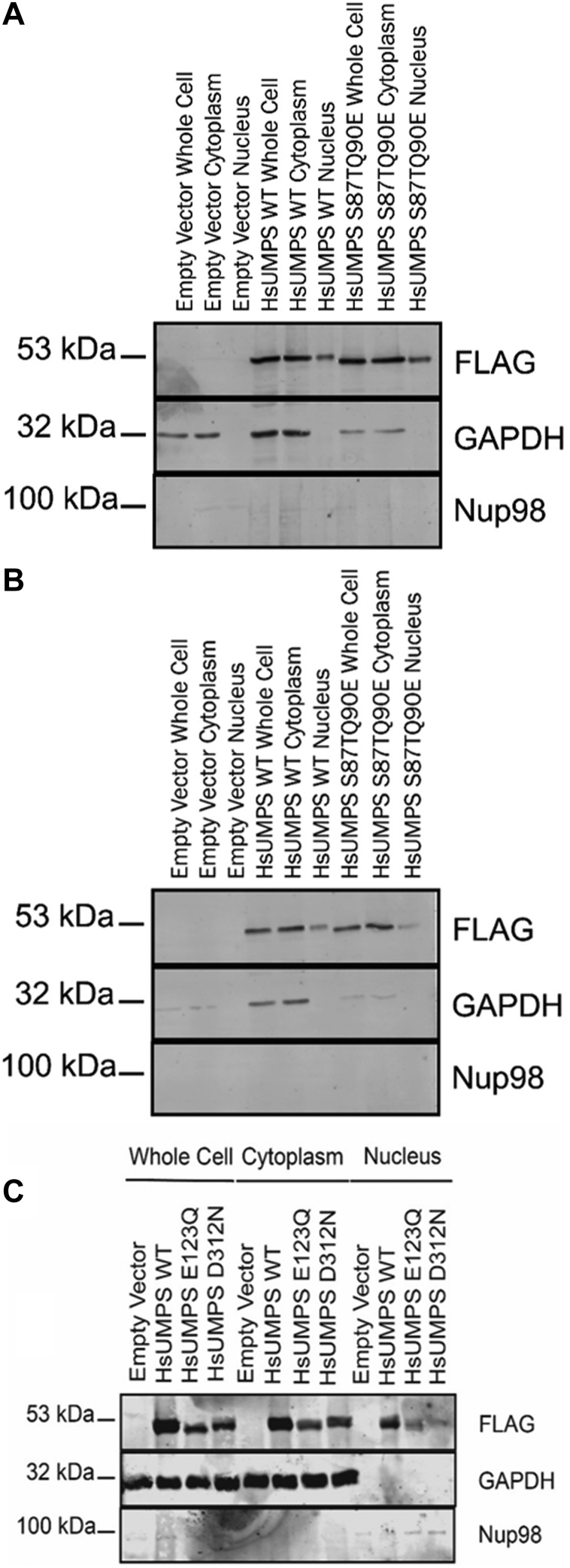
**Cellular localization of HsUMPS.** HeLa cells, transfected with plasmid expressing either flag-tagged native HsUMPS protein (WT) or the flag-tagged oligomerization deficient mutant (S87TQ90E), were fractionated using the REAP approach (see Experimental procedures) to determine the localization of HsUMPS. For both WT and the mutant, HsUMPS appears predominantly in the cytoplasm but also localizes to the nucleus. GAPDH is used for a cytoplasmic marker while Nup98 is used for a nuclear marker. *A* and *B*, the experiments were conducted in either nucleotide rich growth media (*A*) or in nucleotide depleted media (*B*). *C*, the mutant proteins (*C*) show a similar cellular distribution. For all three sets of data, the first three (*A* and *B*) or four (*C*) lanes are for the whole cell fraction, the next three (*A* and *B*) or four (*C*) are for cytoplasmic fraction, and the last three (*A* and *B*) or four (*C*) are for the nuclear fraction. HsUMPS, human UMP synthase; REAP, rapid, efficient, and practical; UMP, uridine 5′-monophosphate.

**Figure 10:**
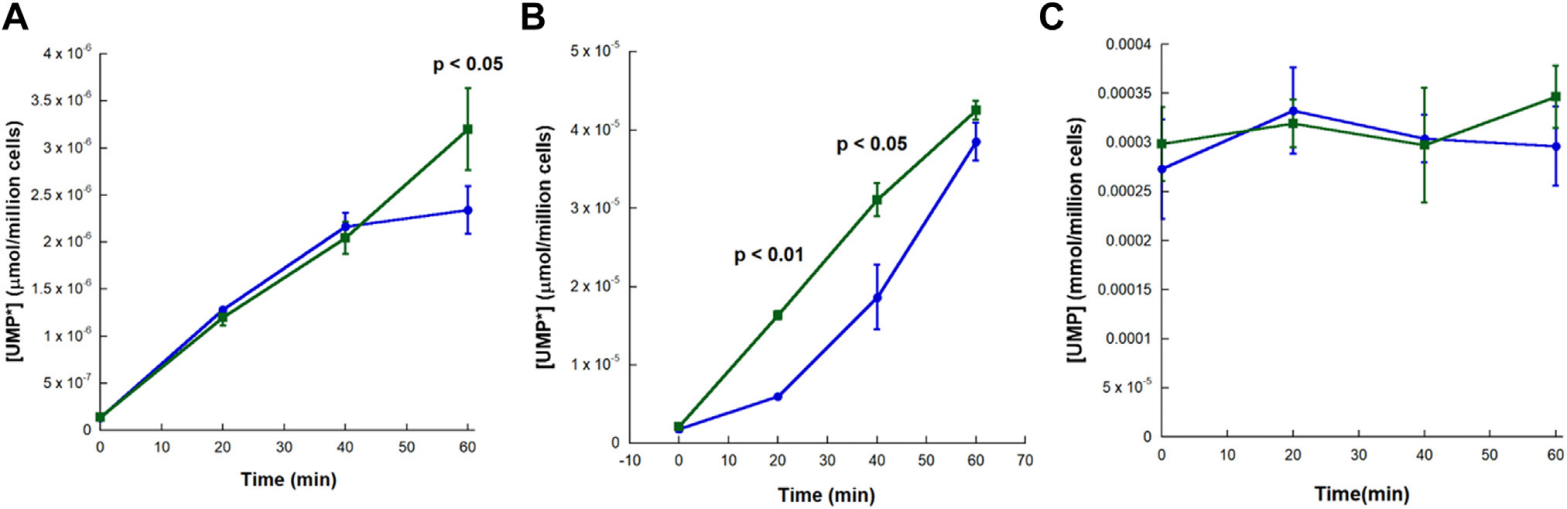
**Rate of UMP biosynthesis in WT and oligomerization deficient mutant HsUMPS.***A* and *B*, LC-MS/MS quantification of isotopically labeled UMP (UMP∗) was used to compare the rate of *de novo* biosynthesis of UMP in rich media (*A*) and nucleotide depleted media (*B*). While the UMP concentrations in cells transfected with plasmid expressing native UMPS (*blue circles* and *line*) level off after time in rich media and take time to reach maximal velocity in depleted media, cells transfected with plasmids harboring the oligomerization-deficient mutant (*green squares* and *line*) appear to have a consistent, high rate of biosynthesis. *C*, as a control, the total UMP concentration (*C*), calculated from a standard curve using an authentic UMP standard, was determined from cells transfected with plasmids containing WT HsUMPS (*blue*) or the oligomerization deficient (S87TQ90E) mutant (*green*) over time. The error bars shown represent the 95% confidence interval, and the *p*-values listed are from independent *t*-tests comparing the two sets of triplicate values at that time point. HsUMPS, human UMP synthase; OPRT, orotate phosphoribosyl transferase; UMP, uridine 5′-monophosphate.

Higher order structures of metabolic proteins can serve a variety of functions in the cells. Some of the most widely accepted roles of these structures include regulation of biosynthetic rates and control of cellular localization (30, 32, 36, 38, 40, 50). To examine possible roles that HsUMPS condensates may play in cells, we first examined the effect that these structures may have on cellular localization and distribution. Using HeLa cells transfected with plasmid encoding flag-tagged HsUMPS, we used immunoblotting to quantify HsUMPS in the nuclear or cytoplasmic fractions under both nucleotide-rich and nucleotide-depleted conditions. The WT HsUMPS was found predominantly in the cytoplasm, although a small fraction could also be observed in the nucleus (Fig. 9). The oligomerization-deficient mutant (UMPS S87TQ90E) and the active site mutants (UMPS E123Q and UMPS D312N) (Fig. 9*C*) also showed a similar cellular distribution. In all cases, the distribution was similar for both nucleotide-rich and nucleotide-depleted conditions. Similar experiments using immunofluorescence to quantify cellular distribution yielded the same results. These also suggest that HsUMPS appears to localize both in the cytoplasm and nucleus and that the oligomerization-deficient mutant does not appear to alter cellular distribution of HsUMPS (Fig. S9). To determine the effect on metabolic flux, we quantified the amount of incorporation of a heavy isotope by mass spectrometry. Cells grown in either nucleotide-rich or nucleotide-depleted media were supplemented with isotopically labeled aspartic acid, and the concentrations of UMP and isotopically labeled UMP (UMP∗) were measured. The amount of UMP∗ produced over time is a direct measure of metabolic flux through the *de novo* pyrimidine biosynthetic pathway as compared to total UMP, which results from biosynthesis *via* both salvage and *de novo* pathways, as well as from transport. Note that while the total amount of UMP∗ produced is a small fraction of total UMP, it is a reliable surrogate to measure UMP biosynthetic rates. We compared the rate of UMP production over time for WT and the oligomerization-deficient mutant HsUMPS in both nucleotide-rich and nucleotide-depleted media. In all cases, the total UMP concentrations remained stable over the course of the experiment (Fig. 10*C*). The production of UMP∗ increased over time, with the cells growing in nucleotide-depleted media showing a metabolic rate nearly an order of magnitude faster than that of cells grown in rich media, as expected (Fig. 10). For the cells grown in rich media (Fig. 10*A*), while the UMP production rate from *de novo* biosynthesis leveled off for the WT UMPS after 40 min to 1 h, the oligomerization-deficient mutant continued to show an increasing rate of UMP synthesis. In nucleotide-depleted media (Fig. 10*B*), both samples trend toward a similar maximal rate of biosynthesis. However, the oligomerization-deficient HsUMPS mutant appears to reach that rate immediately and maintains it throughout the experiment, while the WT HsUMPS has a substantial lag time before reaching a similar rate of flux. Taken together, these data suggest that the HsUMPS mutant that is unable to oligomerize may be compromised in its ability to self-regulate metabolic activity and appears to be constitutively active.

### Model for UMPS regulation of metabolic activity

**Figure 11:**
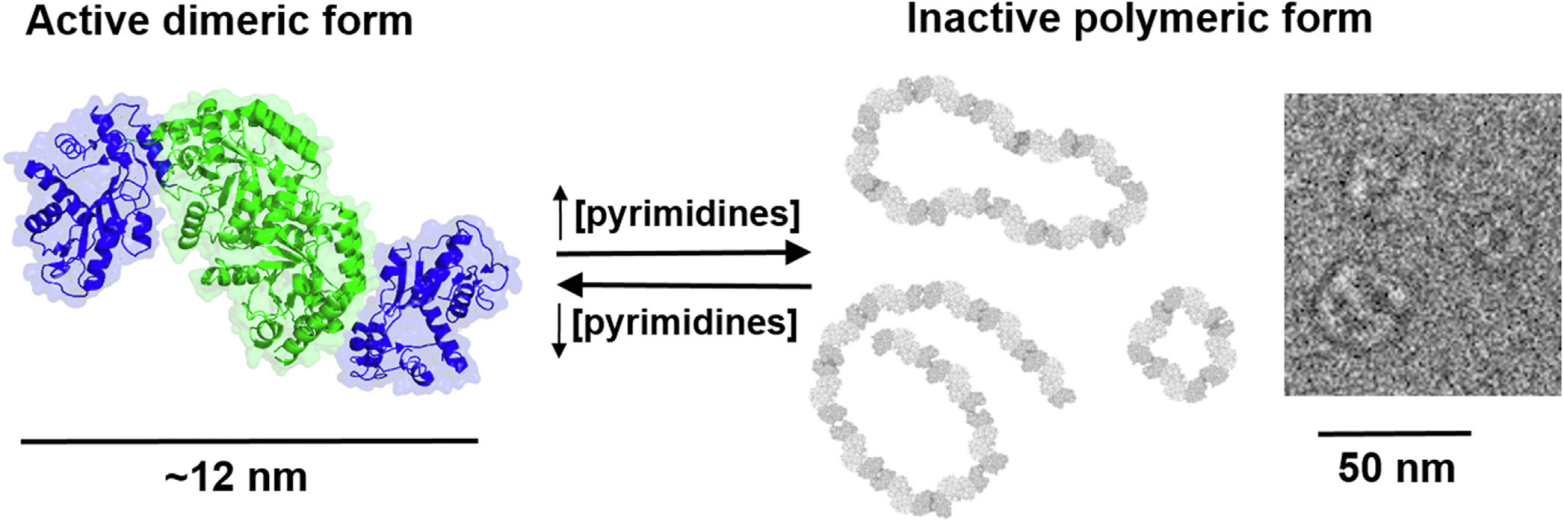
**Proposed model for regulation of HsUMPS activity by substrate-mediated oligomerization.** The dimeric form of HsUMPS has an S-shaped structure and is fully enzymatically active. In the presence of high concentrations of substrates or intermediates, conformational changes in the OPRT domain drive oligomerization of the protein into an inactive polymeric form. As concentrations of the substrate become limiting, the polymers dissociate, creating a pool of active enzyme that can immediately begin generating UMP. HsUMPS, human UMP synthase; OPRT, orotate phosphoribosyl transferase; UMP, uridine 5′-monophosphate.

We have observed that HsUMPS can be found in two distinct structural states that are sensitive to substrate concentrations and perturbations of the active site. The smaller form of the protein is an active dimer, while the larger form is an inactive, polymeric condensate. The relative distribution of the states depends upon substrate concentrations and appears to primarily be driven by features of the OPRT domain. While the localization of the protein is not impacted by the distribution of states, a mutant HsUMPS that does not form the larger oligomeric forms appears to be constitutively active. These observations suggest that HsUMPS may use the differential distribution between structural states as a form of metabolic regulation. In this manner, the larger, inactive state of the protein is a form of storage for metabolic potential. When pyrimidine nucleotide concentrations are sufficient, the protein would be predominantly in the inactive state. When concentrations drop, the equilibrium between higher MW species and lower MW species shifts, liberating active HsUMPS to catalyze the synthesis of UMP. We summarize this proposed model of regulation in Figure 11. Although little is known about the functional implications of structural changes in the human enzyme, OPRT has been observed to undergo conformational changes that drive complex assembly or disassembly and contribute to changes in activity in other eukaryotes (17, 19). In our proposed model, increasing concentrations of orotate or OMP would cause conformational changes of OPRT that favor dimerization and, ultimately, the formation of inactive polymers. A related enzyme in the downstream pyrimidine nucleotide biosynthesis, CTP synthase, has been shown to exhibit a similar polymer-forming regulatory mechanism (32, 33). This type of regulation would provide several obvious advantages. Because this relies upon a simple, concentration-dependent equilibrium, the protein would only be active when needed and could respond to changes in nucleotide concentration very quickly, without the need to synthesize protein. In addition, as observed for other bimolecular condensates, this type of mechanism could also offer spatial regulation. The inactive HsUMPS structures could potentially be trafficked to specific sites throughout the cell to generate high local concentrations of active enzyme.

In some of the first studies of a eukaryotic UMPS, conducted over 4 decades ago, Traut and Jones determined that UMPS, purified from mouse Ehrlich ascite cells, could be observed in multiple different ‘aggregation states’ and that dimerization could be promoted by OMP (22, 45, 51). Here, we have shown that the human protein can freely equilibrate between an active dimeric form and an inactive state that appears to aggregate into a globular polymer. Similar to the original observation for the mouse protein and more recent data in other species, we observed that the transition between these states can be driven by a substrate of the reaction. Taken together, our data suggest that this is a subtle control mechanism for regulating pyrimidine metabolism in the cell by modulating the proportion of active state and, thus, the biosynthetic rate of UMP. The formation of the inactive aggregates may also facilitate transfer of metabolic potential to specific sites within the cell. Since the enzyme that catalyzes the fourth step in the pathway, DHODH, is a mitochondrial enzyme, it is possible that HsUMPS polymers are trafficked to the mitochondria to improve metabolic efficiency when nucleotide levels drop. While further studies will be needed to better understand the extent of this regulation and how it may impact other metabolic processes, it is clear that HsUMPS, like other proteins involved in nucleotide metabolism, has a dynamic structure that can form functionally relevant biomolecular condensates.

## Experimental procedures

### Protein expression and purification

Codon-optimized human UMPS (BioBasic) was subcloned into pDB-His-MBP vector (DNASU), between the NdeI and XhoI restriction endonuclease sites, using standard methods. This construct generates a protein with an N-terminal hexahistidine-tagged MBP with a TEV protease recognition site for proteolytic cleavage postpurification. Overnight cultures of transformed BL21(DE3) cells were grown in Luria Broth (LB) medium supplemented with 50 μg/μl kanamycin at 37 °C. The overnight cultures were used to inoculate 1.0 l of terrific broth medium with 50 μg/μl kanamycin. This culture was grown, with shaking, at 37 °C for approximately 4 to 6 h. When the cells reached an A_600_ value of 1.2 to 1.4, the temperature was decreased to 18 °C. After 1 h, protein expression was induced by the addition of 0.1 mM isopropyl β-d-1-thiogalactopyranoside, and the culture was left to grow for an additional 18 h. Cells were then pelleted at 5000*g* for 15 min at 4 °C and either lysed immediately or stored at −20°C for later purification.

One liter cell pellets were re-suspended in amylose resin binding buffer (20 mM Tris, pH. 8.0, 500 mM NaCl, 1.0 mM EDTA, and 2.0 mM freshly prepared DTT) and lysed by sonication. The cell lysate was clarified by centrifugation at 33,000*g* for 1 h at 4 °C. Cleared lysate was then incubated with pre-equilibrated amylose resin (NEB) rotating for 1 h at room temperature before flowing the lysate with the resin through a gravity column. The resin in the column was then washed with ten column volumes of binding buffer. Protein bound to resin was incubated overnight with 10 μg 6X-His-tagged TEV protease and TEV reaction buffer (50 mM Tris-HCl pH 8.0, 0.5 mM EDTA, 5.0 mM β-mercaptoethanol) while rotating slowly at 4 °C. The protein/resin solution was flowed through a gravity column. Cleaved protein in the flow-through was collected and concentrated with a centrifugal concentrator before buffer exchanging into Ni resin binding buffer (20.0 mM Tris, pH 8.0, 500 mM NaCl, 5.0 mM imidazole) using an Econo-Pac 10DG desalting column (Bio-Rad). The protein solution was applied to a column with pre-equilibrated Ni-NTA agarose resin (Gold Bio) to remove the TEV protease, MBP and remaining undigested protein. The flow-through was collected and further purified by SEC (see below). The protein was determined to be >95% pure by SDS-PAGE. The protein was concentrated on a centrifugal concentrator and the final concentration was determined by Bradford method [14]. The mutant proteins were purified in a similar fashion as WT.

### Size-exclusion chromatography

Purified protein was buffer exchanged into SEC Buffer (20 mM Tris pH 7.5, 100 mM NaCl) using an Econo-Pac 10DG desalting column (Bio-Rad) and concentrated with a 10,000 MWCO centrifugal concentrator for further purification by SEC. This step involved purifying protein on an AKTA FPLC with SEPAX SRT-10-SEC-300, GE Superdex 200 10/300 or GE Superdex 200 16/600 columns. Eluted protein from SEC was concentrated with a centrifugal concentrator and aliquoted in 50 μl aliquots. Aliquots were either used immediately after purification or snap frozen in liquid nitrogen and stored at −80 °C.

### Anion exchange chromatography

Digested protein collected from Ni-NTA flowthrough was buffer exchanged into AEX Buffer A (50 mM Tris pH 8.0) and diluted to 50 ml to be further purified by AEX. This step involved purifying protein on an AKTA FPLC with GE 5.0 ml HiTrap Q HP column with AEX Buffer A (50 mM Tris pH 8.0) and AEX Buffer B (50 mM Tris pH 8.0, 1.0 M NaCl). After loading the sample, the column was washed with 50 ml 3% AEX Buffer B before a stepwise elution of 15% AEX Buffer B and 30% AEX Buffer B. Eluted protein was buffer exchanged into storage buffer (20 mM Tris pH 7.5, 100 mM NaCl) before snap freezing in liquid nitrogen and stored at −80 °C.

### Site-directed mutagenesis

Site-directed mutagenesis experiments were performed with a Q5 Mutagenesis kit (New England Biolabs) following the manufacturer’s protocol. All constructs generated by mutagenesis were confirmed with Sanger DNA sequencing (GenScript).

### Enzyme kinetics

The enzyme kinetics of HsUMPS enzyme were monitored by absorbance changes over time at 279 nm for the production of UMP and at 295 nm for the consumption of orotate. These reactions were conducted at 25 °C on a Synergy Neo2 microplate reader (BioTek) or SpectraMax M2 (Molecular Devices). The standard assay conditions contained 50 mM Tris pH 8.0, 100 mM NaCl, 2 mM MgCl_2_. For kinetic assays, the mixture consisted of either 100 μM PrPP with varying concentrations of orotate (0–200 μM) or varying concentrations of OMP (0–200 μM). In all cases, the reaction was initiated by the addition of protein (5–50 pmol). Absorbance data were collected in triplicate, averaged, and plotted as mean ± standard error. To calculate kinetic parameters, initial rate date (before 10% reaction completion) was fit with the Michaelis–Menten equation.

### Electron microscopy

For negative stain images, 3 μl of sample at a concentration of 350 μg/ml was placed on a 400-mesh copper grid coated with a formvar/carbon film (Electron Microscopy Sciences) for 1 min before washing three times with 5 μl distilled water. Excess water was removed with Whatman filter paper. The sample was negatively stained with 3 μl UranyLess (EMS) for 30 s. Excess stain solution was removed with Whatman filter paper, before the grid was slowly dried at room temperature. Electron micrographs were taken with a FEI Tecnai Biotwin Spirit transmission electron microscope at an accelerating voltage of 120 kV.

Specimens for single particle cryo-EM were prepared by applying 4 μl of 0.6 mg/ml protein to glow-discharged Quantifoil Cu R1.2/1.3 grids (Electron Microscopy Sciences). The grids were blotted for 5 s at room temperature under 95% humidity and plunge frozen in liquid ethane using a Vitrobot Mark IV (Thermo). Cryo-EM data were collected using a Titan Krios microscope operating at 300 kV equipped with a Falcon 3EC direct electron detector (Thermo). Micrograph movies were recorded in counting mode using the EPU automated data collection software at nominal magnification of 96,000×, which corresponds to a pixel size of 0.895 Å. All data processing, including motion correction, CTF estimation, automatic particle picking, and 2D classification, were carried out using Relion 3.1 (52).

### Analytical ultracentrifugation

Four hundred microliter samples were prepared at concentrations of 1 mg/ml (WT) or 1.5 mg/ml (mutants) alongside 1:5 dilutions in the buffer originally used for purification. Samples were loaded into cells with quartz windows and Epon charcoal filled centerpieces. Using a Beckman Optima XL-A centrifuge and an An-60 Ti rotor at 38,000 rpm, absorbance traces were collected at 280 nm until no further change in sedimentation traces over time was observed. Following data export using UltraScan III, traces were analyzed with SEDFIT to calculate continuous sedimentation profiles and the corresponding MW of the primary resulting peaks. Due to the observed heterogeneity of the WT protein, the sedimentation ratio of the WT dimer was held constant at that of the mutant dimers to enable analysis.

### Aldehyde crosslinking oligomerization analysis by SDS-PAGE

The protein was buffer exchanged into 50.0 mM Na_3_PO_4_ pH 8.0, 100.0 mM NaCl and purified with SEC. The different SEC fractions were collected and crosslinked with a final concentration of 0.004% glutaraldehyde. Samples were quenched at select time points with SDS buffer and 1.0 M Tris pH 8.0 before visualization by SDS-PAGE.

### LC/MS quantification of pyrimidine nucleotides

Pyrimidine biosynthetic rates were measured using a modification of a method developed to determine purine biosynthetic rates (36). Low passage number HEK293T cells were maintained in either standard (purine rich) media, DMEM (Corning) with 10% fetal bovine serum and 100 U/ml pen/strep (Gibco), or purine depleted media (28, 36, 38), consisting of DMEM (Corning) supplemented with 10% dialyzed fetal bovine serum and 100 U/ml pen/strep, for at least three passages. Cells were transfected with plasmid containing either native or oligomerization-deficient mutant (S87TQ90E) HsUMPS in a p3xflag-Myc_CMV vector using PEI (Sigma Aldrich) following the manufacturer’s suggested protocol. Western blotting was used to confirm that expression levels of WT or mutant HsUMPS were equivalent (Fig. S10). At 36 h, posttransfection cells were transferred to fresh media containing 500 μM ^13^C4,^15^N-aspartic acid (Cambridge Isotope Labs) for the desired interval (0, 20, 40, or 60 min) before being washed three times with ice-cold PBS and harvesting with 0.05% trypsin-EDTA (Gibco).

Cells were lysed using RIPA buffer (20 mM Tris, 15 mM NaCl, 1 mM EDTA, 1 mM DTT, and 1% Triton X-100) supplemented with protease inhibitor cocktail (Sigma Aldrich) for 30 min on ice with occasional vortexing. Following incubation, cell suspensions were centrifuged at 13,000*g* for 15 min, and the supernatants were carefully transferred to a 3000 MW cutoff centrifugal filter. Samples were centrifuged at 14,000*g* for 25 min and the flow-through was analyzed by LC-MS/MS to quantify UMP and UMP∗ levels. Note that, for total UMP concentration, samples were diluted up to 1000-fold before measurement to ensure they were in the linear range of the standard curve used to determine concentration.

The liquid chromatography-mass spectrometry analysis was performed using a 6460C triple quad mass spectrometer (Agilent) with an inline 1290 LC system (Agilent) using an approach adapted from prior studies (41, 53). Chromatographic separation of UMP from other nucleotides was achieved using hydrophobic interaction LC using a 2.1 × 100 mm, 2.7 micron Poroshell 120 HILIC-Z column (Agilent) at 30 °C. A gradient elution using 10% acetonitrile (buffer A) and 90% acetonitrile (buffer B) both in 10 mM ammonium acetate, pH 5.1, at 0.25 ml/min was employed as follows: 0% buffer B to 3 min, linear gradient to 40% buffer B to 20 min, isocratic at 40% buffer B to 25 min, gradient to 0% to 30 min, and isocratic at 0% buffer B to 35 min. The mass spectrometer was run using multiple reaction monitoring in negative ion mode using nitrogen as a nebulizing gas. The experimental conditions were set as follows: ion source temperature, 250 °C; sheath gas temperature, 350 °C; ion source gas flow and sheath gas flow, 11 l/min; nebulizer pressure, 45 psi; nozzle voltage, 1.5 kV; and capillary current, 3500 nA. The conditions of the multiple reaction monitoring transitions were as follows [fragmenter voltage (V), collision energy (eV)]: UMP, 327-97 (127, 21); UMP, 327-115 (127, 25); UMP, 327-79 (127, 40); UMP∗, 327-97 (127,21); UMP∗, 327-111 (127,25); UMP∗, 327-79 (127, 40). UMP and UMP∗ were quantified by integrating the peak area with Agilent QQQ Quantitative Analysis (v 10.1) and using a standard curve derived from an authentic standard of UMP-disodium salt (Alfa Aesar).

### Cellular fractionation and analysis

The rapid, efficient, and practical method was used to isolate whole cell, cytoplasmic, and nuclear fractions according to the published protocol [18]. Transfected HeLa cells were cultured as monolayers in 10 cm diameter dishes. Cells were first washed three times with 10 ml ice-cold PBS, pH 7.4, which was then removed by aspiration. 1 ml ice cold PBS was added to the plate, and cells were scraped from culture dishes with a cell scraper and collected in 1.5 ml microcentrifuge tubes. “Pop spin” centrifugation for 10 s was done in a tabletop microcentrifuge at 12,000*g*. The supernatant was removed, and cells were re-suspended with ice-cold 0.1% (vol/vol) NP-40 (Boston Bioproducts) in PBS. 300 μl of lysate was saved as whole cell lysate, to which 100 μl of 4× Laemmli sample buffer was added and kept on ice until the sonication step. The remaining lysate was centrifuged for 10 s, and 300 μl of the supernatant was removed as the cytosolic fraction. 100 μl of 4× Laemmli was added to this fraction and boiled for 1 min. The remaining supernatant was removed, and the pellet was re-suspended in 1 ml ice-cold 0.1% (vol/vol) NP-40 in PBS and centrifuged for 10 s. The supernatant was discarded, and the remaining pellet was re-suspended with 180 μl 1× Laemmli sample buffer and kept as the nuclear fraction. Nuclear and whole cell fractions were sonicated two times using a microprobe, for 8 s each, followed by boiling for 1 min. FLAG-tagged protein was detected in the whole cell, cytoplasmic, and nuclear fractions with SDS-PAGE electrophoresis followed by immunoblotting. Transferred membrane (Immobilon-P, Merck) was incubated with FLAG antibody (Sigma) followed by IRDye 680RD Goat anti-mouse immunoglobulin G (IgG). The membrane was imaged with a Licor Odyssey CLx imaging system. The membrane was then stripped with mild stripping buffer and similarly probed with GAPDH (Santa Cruz or Invitrogen) and β-tubulin antibody (Sigma)as a cytoplasmic marker and Nup98 antibody (Santa Cruz Biotechnology) as a nuclear marker.

## Data availability

The majority of the data are provided and described in the manuscript and in the supporting information files. Raw and processed SAXS data are available for download as supplemental data. Other raw data, such as cryo-EM movies, are available upon request from the corresponding author.

## Supporting information

This article contains supporting information.

## Conflict of interest

The authors declare no conflict of interest with the contents of this article.

## Supporting information

Supporting Figures S1–S10

SAXS
