# Supplementary material for: Human uridine 5′-monophosphate synthase stores metabolic potential in inactive biomolecular condensates
Source: J Biol Chem. 2023 Jan 25;299(3):102949. doi: 10.1016/j.jbc.2023.102949 (PMC9978035; doi:10.1016/j.jbc.2023.102949)
Supplement: Supporting Figures S1–S10 [file mmc1.docx]

**Supporting Information**

**Human Uridine 5′-Monophosphate Synthase Stores Metabolic Potential in Inactive Biomolecular Condensates**

Deborah M. Kim-Holzapfel^1,2^, Raja Dey^3^, Brian C. Richardson^3^, Danushka Arachchige^3^, Kanamata Reddy^3^, Humberto De Vitto^3^, Janarjan Bhandari^3^, and Jarrod B. French^3,^*

^1^ *Department of Biochemistry and Cell Biology, Stony Brook University, Stony Brook, NY 11794*

^2^ *Molecular and Cellular Biology PhD Program, Stony Brook University, Stony Brook, NY 11794*

^3^ *The Hormel Institute, University of Minnesota, Austin, MN 55912*

*To whom correspondence should be addressed:

Jarrod B. French

jfrench@umn.edu

(507) 437-9637

Figure S1. Steady state kinetics of HsUMPS domains

Figure S2. Steady state kinetics of mutant forms of HsUMPS

Figure S3. Molecular weight standards for size-exclusion chromatography.

Figure S4. Enzyme kinetics of dimerization deficient mutant

Figure S5. Size exclusion chromatography of S87T/Q90E mutant

Figure S6. Analytical ultracentrifugation analysis

Figure S7. Additional 2D classes showing S-shaped HsUMPS dimer structure

Figure S8. SEC-SAXS analysis of HsUMPS

Figure S9. Localization of UMPS using immunofluorescence

Figure S10. Expression level of HsUMPS in transfected cells


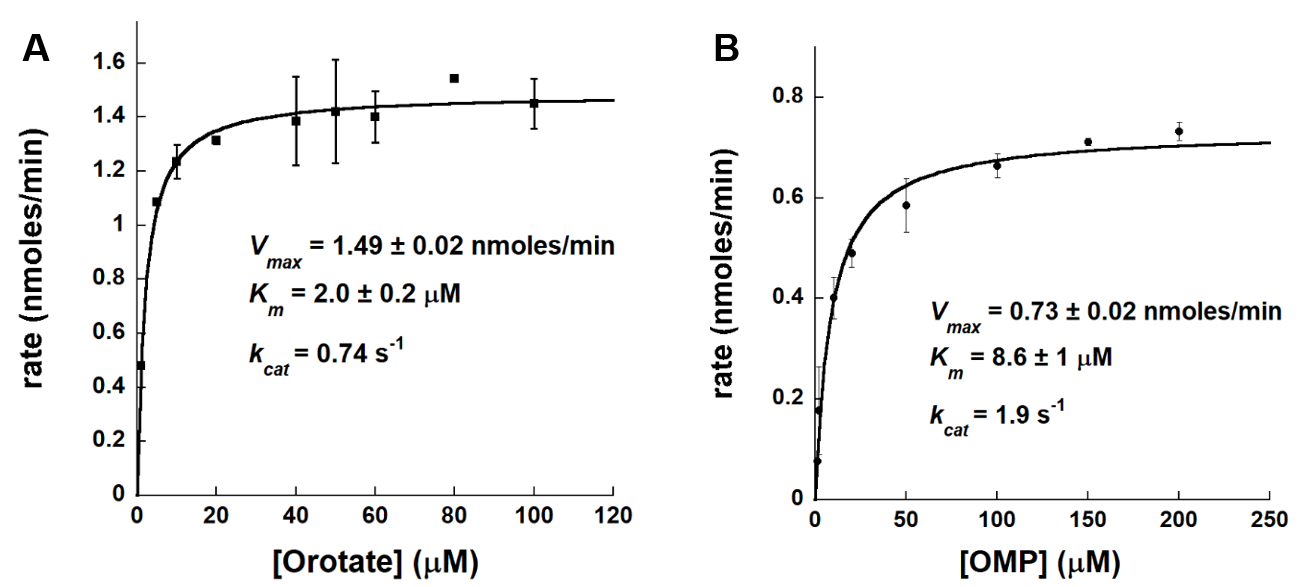


**Figure S1.** Steady state kinetics of HsUMPS domains. The initial rates of the OPRT-catalyzed reaction (A) were measured at 295 nm at various concentrations of orotate and fit with the Michaelis-Menten equation. Similarly, the initial rates of the OMPDC-catalyzed reaction (B) were measured at 279 nm and fit with the Michaelis-Menten equation. The values plotted are averages of triplicate measurements ± standard error.

**
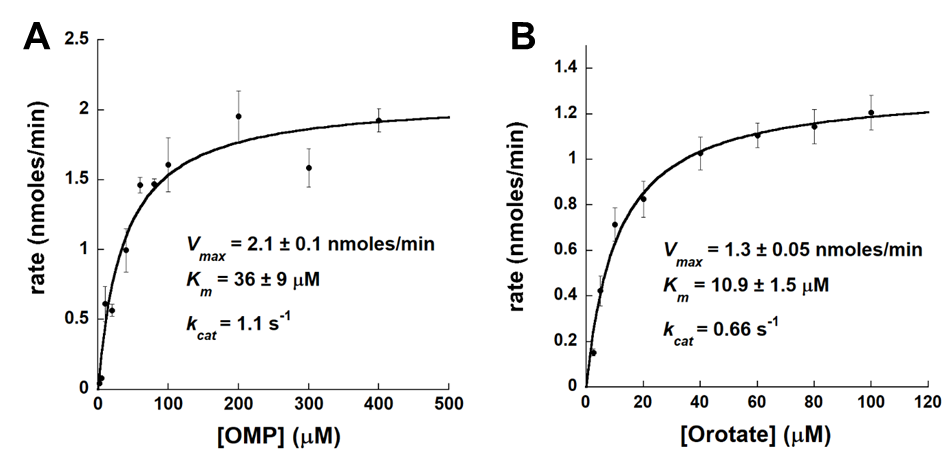
**

**Figure S2.** Steady state kinetics of mutant forms of HsUMPS. The initial rates of the OMPDC-catalyzed reaction (A) were measured at 295 nm for a range of OMP concentrations before being fitted by the Michaelis Menten equation for the E123Q, OPRT-deficient mutant of HsUMPS. There was no observable turnover for the OPRT-catalyzed reaction for this mutant. Similarly, the initial rates of the OPRT-catalyzed reaction (B) were measured at 279 nm for a range of orotate concentrations before being fitted by the Michaelis Menten equation for the D312N, OMPDC-deficient mutant of HsUMPS. There was no observable turnover for the OMPDC-catalyzed reaction for this mutant. The values plotted are averages of triplicate measurements ± standard error.


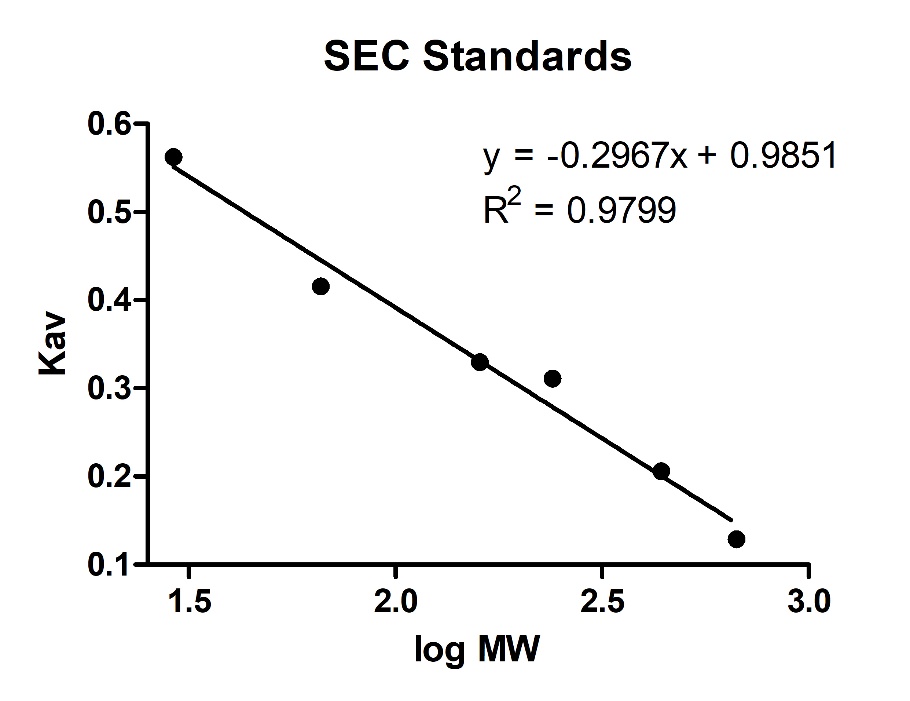


**Figure S3.** Molecular weight standards for size-exclusion chromatography. The standards included thyroglobulin (669 kDa), ferritin (440 kDa), catalase (240 kDa), aldolase (160 kDa), bovine serum albumin (66 kDa), and carbonic anhydrase (29 kDa).


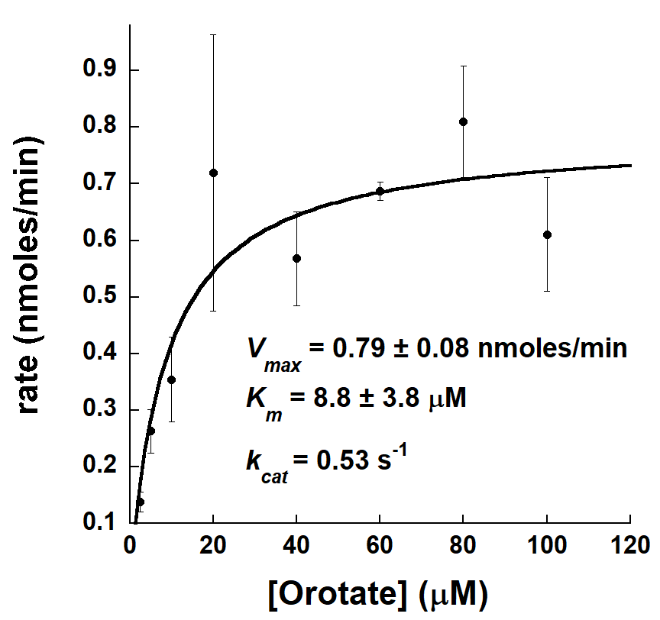


**B**

**A**


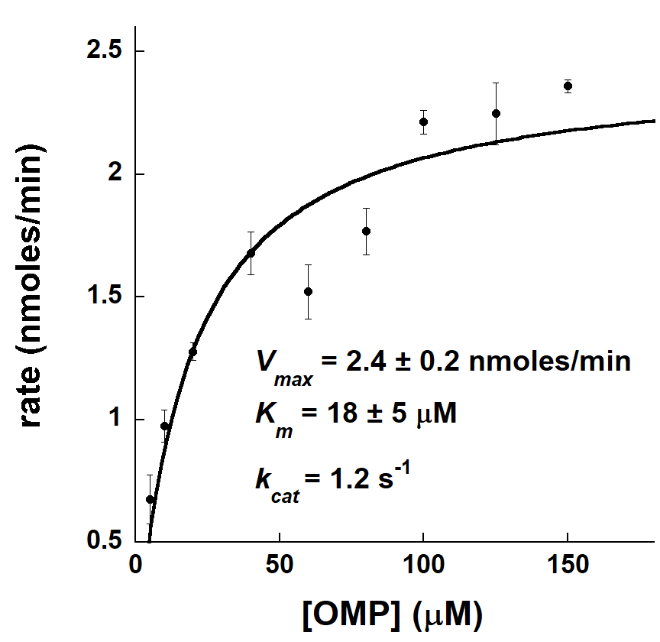
**Figure S4.** Steady state kinetics of the dimerization deficient mutant forms of HsUMPS. The initial rates of the OMPDC-catalyzed reaction (A) were measured at 295 nm for a range of OMP concentrations before being fitted by the Michaelis Menten equation while the initial rates of the OPRT-catalyzed reaction (B) were measured at 279 nm for a range of orotate concentrations before being fitted by the Michaelis Menten equation. The values plotted are averages of triplicate measurements ± standard error.


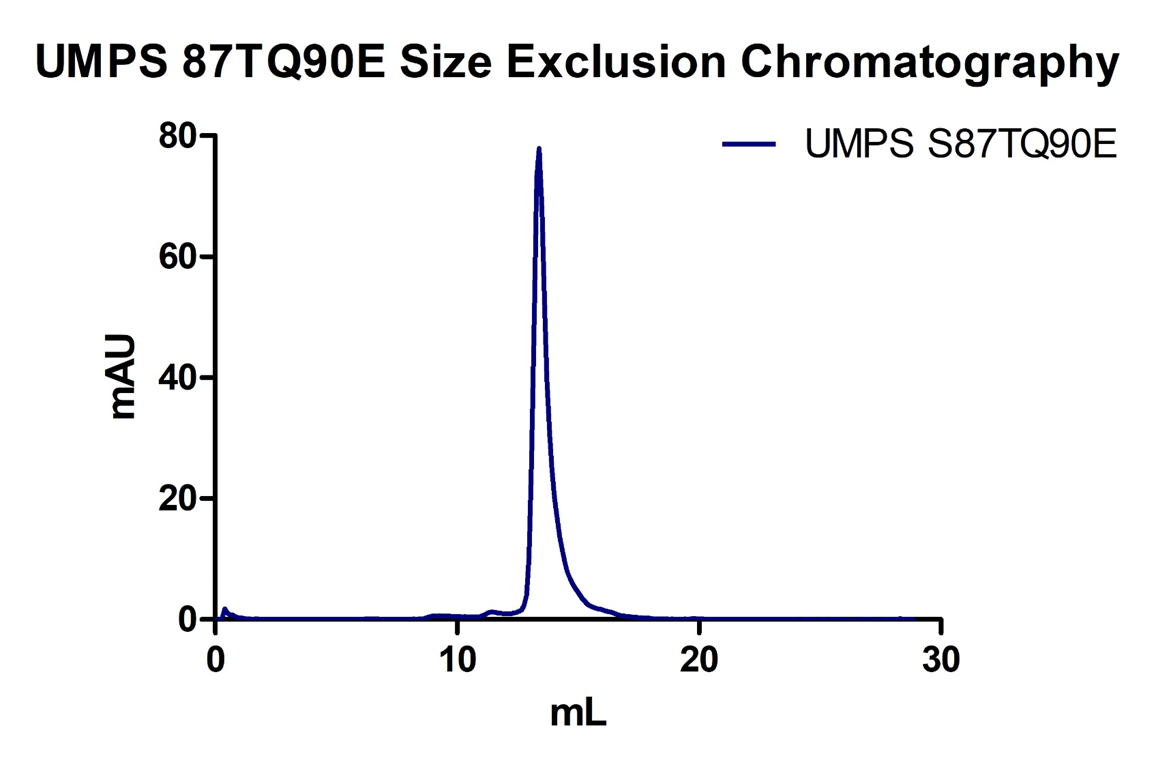


**Figure S5.** Size exclusion chromatography of S87T/Q90E mutant. The HsUMPS OPRT dimerization-deficient mutant was purified and analyzed by SEC using the same conditions as the native protein (see Materials and Methods).


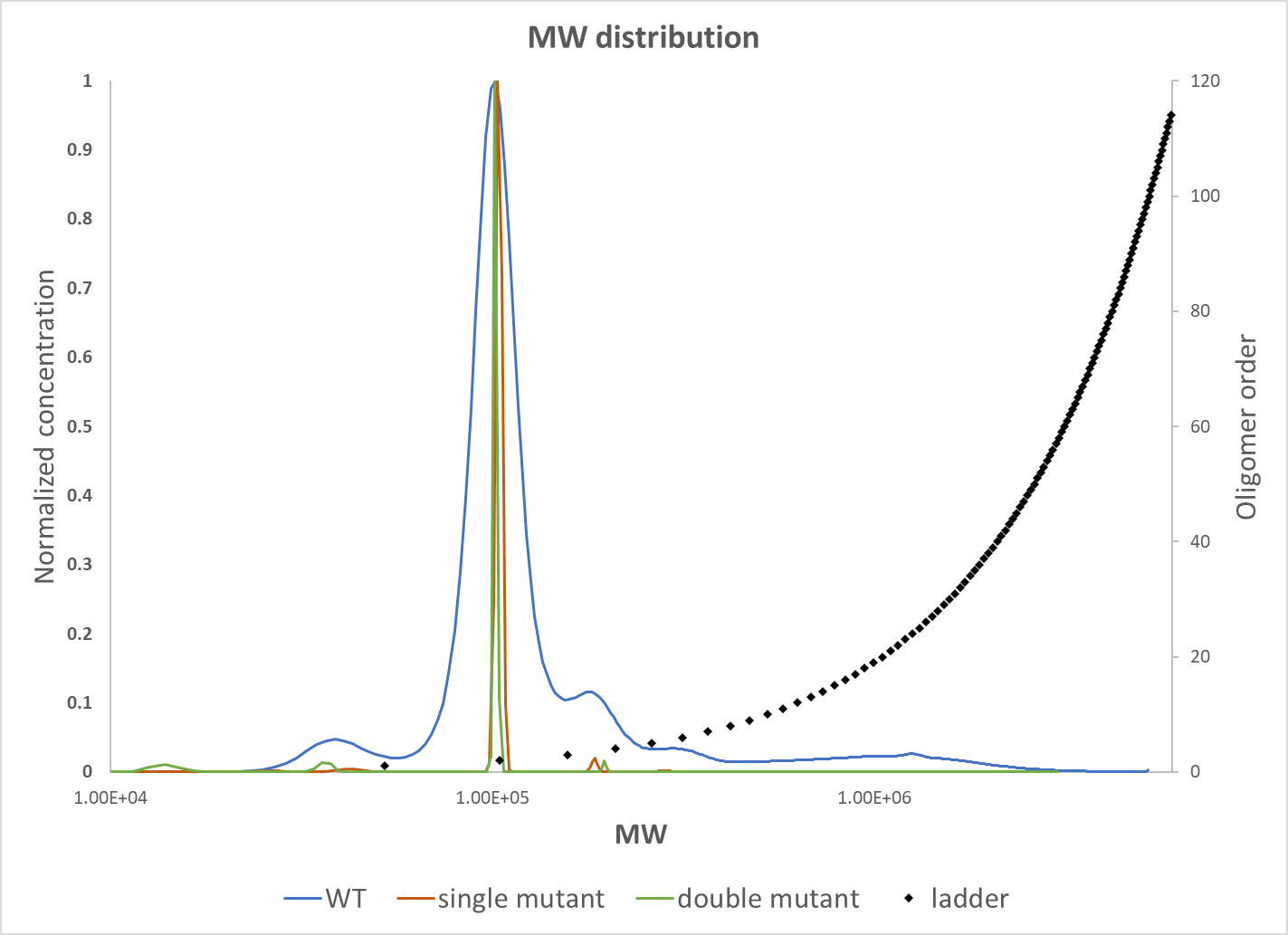


**Figure S6**. Analytical ultracentrifugation analysis. Both the OPRT active site mutant (E123Q, single mutant, orange line) and the OPRT oligomerization-deficient double mutant (S87T/Q90E, double mutant, green line) show sharp peaks corresponding to molecular weights of 102 kDa and 104 kDa, respectively, consistent with a HsUMPS dimer. The native enzyme (WT, blue line) has a broader peak at a molecular weight consistent with a dimer and the presence of additional, smaller peaks, at higher molecular weights.


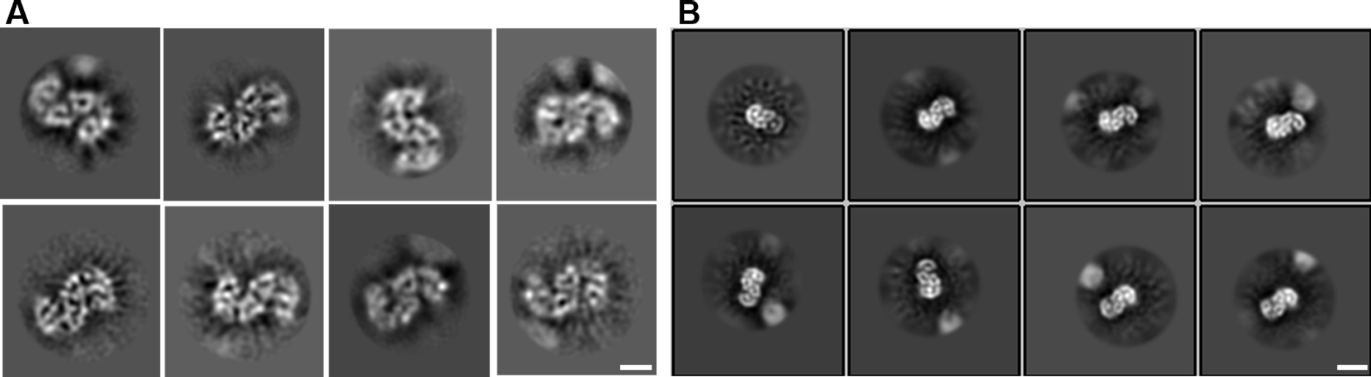


**Figure S7.** Additional 2D classes showing S-shaped HsUMPS dimer structure after initial processing with Relion (A) and CisTem (B). This was from data collected at 120,000X magnification on a Titan Krios. Note that all of these classes represent ~1000-3000 particles each in A and 10,000-20,000 particles each in B. The scale bar in (A) is 30 Å and in (B) is 50 Å.


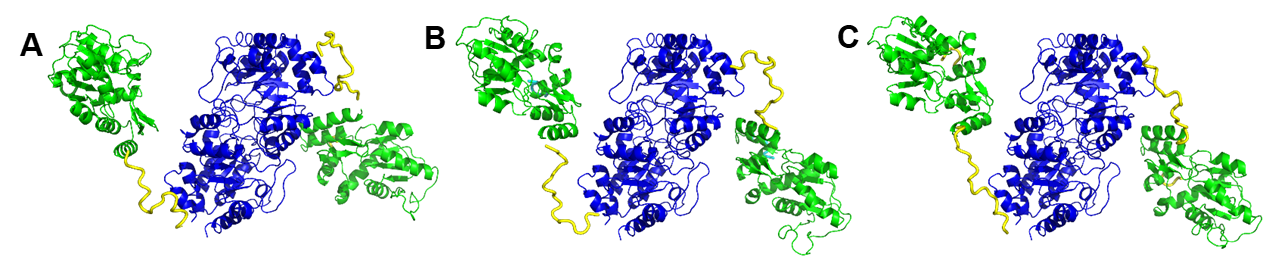


**Figure S8.** Size exclusion chromatography small angle X-ray scattering analysis (SEC-SAXS) of HsUMPS. The ATSAS suite was used to generate models from the scattering data. Models generated using CORAL are shown. In all cases, 3BVJ and 2WNS were used as models for the OMPDC and OPRT domains, respectively. The models were generated either without imposing symmetry across the dimer (A), or with P2 symmetry imposed (B and C) along the OMPDC dimer axis. The model in B uses all of the data, where the model shown in C was generated from a dataset where several low-angle points were discarded during processing by GNOM.


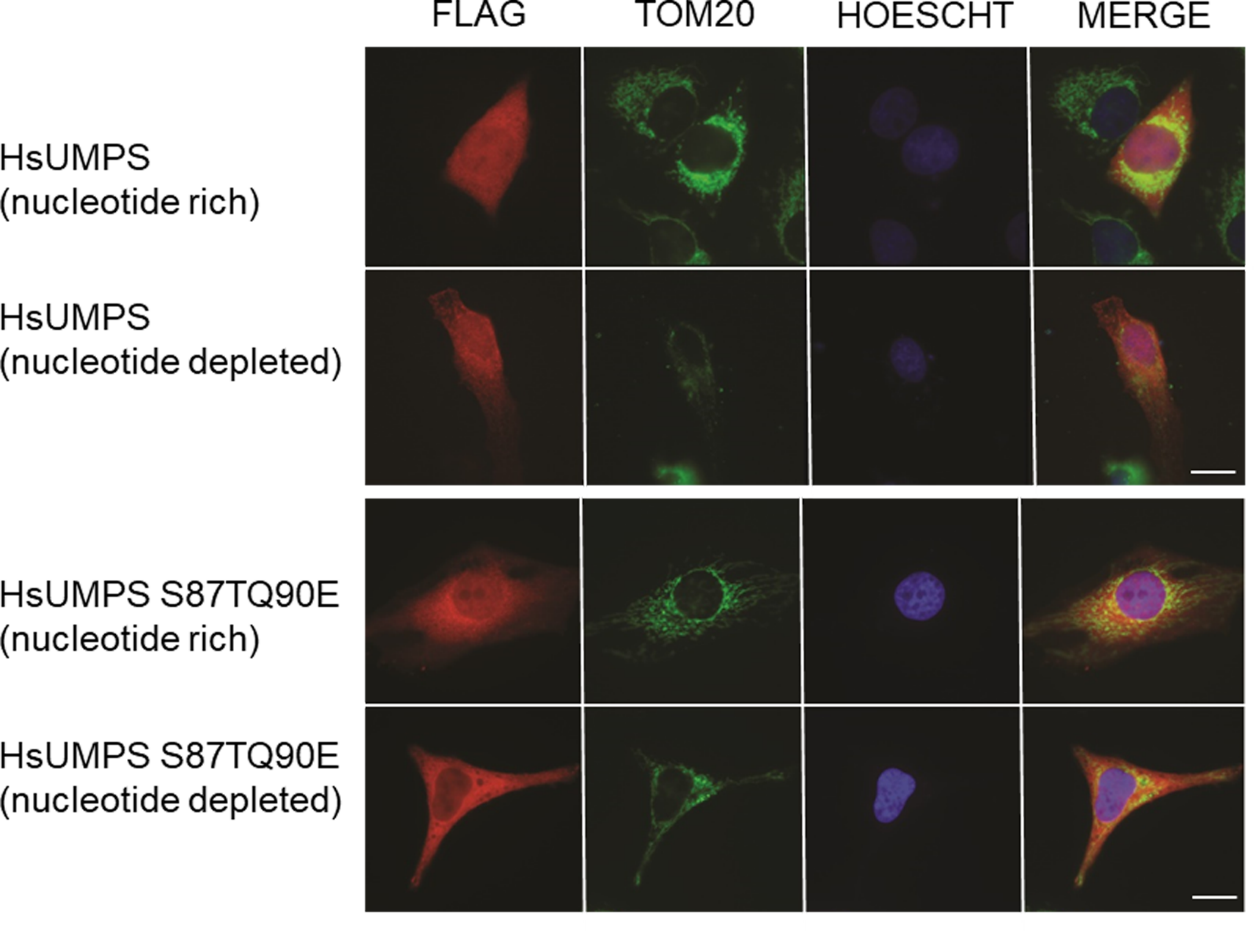


**Figure S9.** Localization of UMPS using immunofluorescence. Representative images (all collected at 60x magnification on a Leica fluorescent deconvolution microscope) of HeLa cells transfected with either HsUMPS-flag vector (top two rows) or HsUMPS S87TQ90E-flag vector (bottom two rows). After fixation, the cells were blocked with blocking buffer, incubated with anti-FLAG mouse antibody (Sigma) and anti-TOM20 rabbit antibody, washed three times, then incubated with goat anti-mouse antibody conjugated with AlexaFluor 594 and donkey anti-rabbit antibody conjugated with AlexaFluor 488 (Jackson ImmunoResearch). During the final washing step, Hoescht was also added. The images in the first column were taken using the Texas Red filter (560nm/630 nm) and illustrate the broad distribution of UMPS in the cytoplasm and nucleus. The second and third columns were taken using the GFP (485 nm/530 nm) and DAPI (355 nm/460 nm) filters, respectively, and show the location of the mitochondria and nucleus. The scale bar is 10 µm.

**
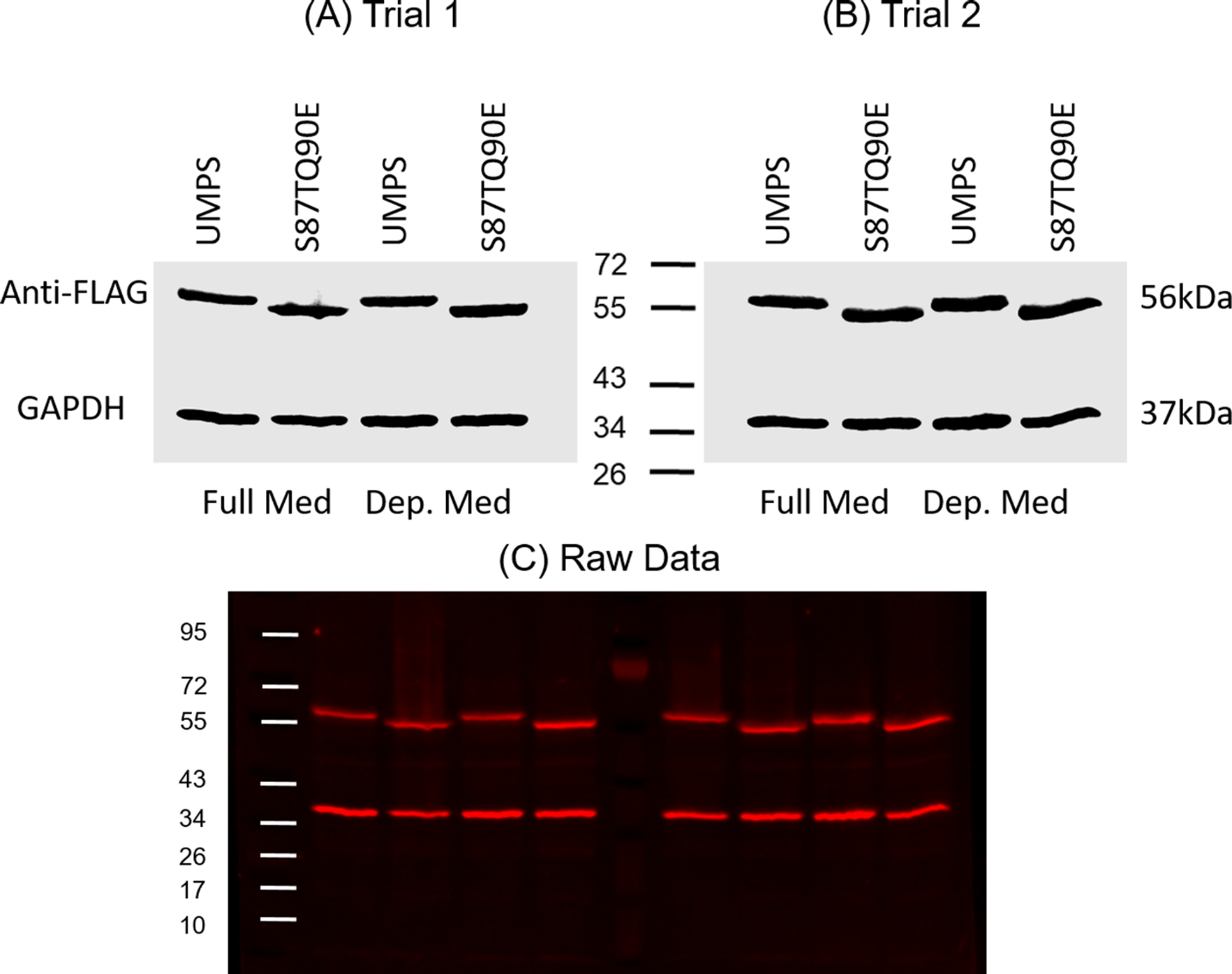
**

**Figure S10.** Expression level of HsUMPS in transfected cells used in MS quantitation of UMP. Two independent samples of cells after transfection with either flag-tagged HsUMPS or flag-tagged HsUMPS S87TQ90E were lysed and analyzed by Western blotting to quantify protein expression levels. An anti-flag antibody (Sigma) was used to label the UMPS while an anti-GAPDH antibody was used as a loading control. (A) and (B) show the inverted contrast-corrected images of trial 1 and trial 2, while (C) shows the raw data. Molecular weight markers are indicated with black or white dashes and the corresponding molecular weights (in kDa) are shown.
